# *Arabidopsis* bZIP18 and bZIP52 Accumulate in Nuclei Following Heat Stress where They Regulate the Expression of a Similar Set of Genes

**DOI:** 10.3390/ijms22020530

**Published:** 2021-01-07

**Authors:** Anna J. Wiese, Lenka Steinbachová, Ljudmilla Timofejeva, Vojtěch Čermák, Božena Klodová, Ranjani S. Ganji, Mariana Limones-Mendez, Pavel Bokvaj, Said Hafidh, David Potěšil, David Honys

**Affiliations:** 1 Laboratory of Pollen Biology, Institute of Experimental Botany of the Czech Academy of Sciences, Rozvojová 263, 165 02 Prague 6, Czech Republic; wiese@ueb.cas.cz (A.J.W.); steinbachova@ueb.cas.cz (L.S.); timofejeva@ueb.cas.cz (L.T.); klodova@ueb.cas.cz (B.K.); limones@ueb.cas.cz (M.L.-M.); pavel.bokvaj@gmail.com (P.B.); hafidh@ueb.cas.cz (S.H); 2 Department of Experimental Plant Biology, Faculty of Science, Charles University, Viničná 5, 128 44 Prague 2, Czech Republic; vojta.ce@gmail.com; 3 Mendel Centre for Plant Genomics and Proteomics, Central European Institute of Technology, Masaryk University, Kamenice 753/5, 62 500 Brno, Czech Republic; ranjani.ganji@ceitec.muni.cz (R.S.G.); david.potesil@ceitec.muni.cz (D.P.)

**Keywords:** bZIP, heat stress, *Arabidopsis*, 14–3–3, localization, transcriptomics

## Abstract

Heat stress (HS) is a major abiotic stress that negatively impacts crop yields across the globe. Plants respond to elevated temperatures by changing gene expression, mediated by transcription factors (TFs) functioning to enhance HS tolerance. The involvement of Group I bZIP TFs in the heat stress response (HSR) is not known. In this study, bZIP18 and bZIP52 were investigated for their possible role in the HSR. Localization experiments revealed their nuclear accumulation following heat stress, which was found to be triggered by dephosphorylation. Both TFs were found to possess two motifs containing serine residues that are candidates for phosphorylation. These motifs are recognized by 14–3–3 proteins, and bZIP18 and bZIP52 were found to bind 14–3–3 ε, the interaction of which sequesters them to the cytoplasm. Mutation of both residues abolished 14–3–3 ε interaction and led to a strict nuclear localization for both TFs. RNA-seq analysis revealed coordinated downregulation of several metabolic pathways including energy metabolism and translation, and upregulation of numerous lncRNAs in particular. These results support the idea that bZIP18 and bZIP52 are sequestered to the cytoplasm under control conditions, and that heat stress leads to their re-localization to nuclei, where they jointly regulate gene expression.

## 1. Introduction

Crop plants have a temperature window within which they grow optimally, with each window having a minimum, optimum, and maximum temperature. Optimal temperatures differ between developmental stages for the same plant, and between different crop species. When the maximum temperature in this window is exceeded, it can have adverse effects on plant growth and development [1]. Due to global warming, the earths’ surface temperature is steadily increasing, which can offset the maximum temperature for a variety of crop plants [2,3]. This in turn has negative impacts on crop productivity, and ultimately food security. A report released by the Intergovernmental Panel on Climate Change in 2013 estimated that the earths’ global temperature is set to increase between 2.6–4.8 °C by the end of the century, as a worst case scenario (http://www.climatechange2013.org) [4]. With such a bleak outlook on future climate scenarios, researchers are tasked with creating crop varieties that are superior in their ability to deal with growth under elevated temperatures. In order to do this, basic research into the molecular mechanisms employed by plants to survive under elevated temperature is necessary. The sessile nature of plants precludes them from moving away from unfavorable conditions, forcing them to deal with elevated temperatures on site. Heat stress (HS) affects virtually every aspect of plant growth and development, ranging from seed germination to reproduction [5]. At the molecular level, it leads to protein unfolding and denaturation, membrane destabilization, and the accumulation of reactive oxygen species [5,6]. To avoid this, plants rapidly set in motion the cytoplasmic heat stress response (HSR) to regain and maintain cellular homeostasis under HS. Central to the HSR are heat shock transcription factors (HSFs), which lead to the expression and accumulation of heat shock proteins (HSPs) that function as chaperones to stabilize proteins under heat stress.

Apart from HSFs, other transcription factors (TFs) have also been shown to respond to elevated temperatures, with some contributing to heat stress tolerance in plants. Among these are certain members of the basic (region) leucine zipper (bZIP) TFs family. bZIP TFs are evolutionarily conserved TFs found to play a role in energy metabolism [7], unfolded protein response [8], senescence [9], flowering [10], pollen development [11,12,13], seed maturation [14], and abiotic stress signaling [15]. These TFs possess a basic DNA-binding domain and a leucine zipper that enables bZIP dimerization. The bZIP family in *Arabidopsis* comprise 78 members, differentiated into 13 groups [16]. As it relates to the stress response, bZIPs from Group B (bZIP17, bZIP28) and K (bZIP60) have been implicated in the HSR. When mis- or unfolded proteins accumulate in the endoplasmic reticulum (ER) under heat stress, the unfolded protein response (UPR) is activated, which upregulates the expression of genes involved in ER protein import, folding, quality control, and export [17]. The TFs responsible for the expression of UPR-associated genes, bZIP60, bZIP17, and bZIP28, are membrane-tethered, sequestering them away from the nucleus under non-stress conditions. In the case of bZIP60, its messenger RNA is spliced by an ER membrane-associated RNA splicing factor, INOSITOL REQUIRING ENZYME 1 (IRE1) [18,19]. The splicing and subsequent translation of *bZIP60* mRNA allows bZIP60 translocation to the nucleus. bZIP17 and bZIP28 are normally retained in the ER through their association with the luminal BiP protein [20]. Following stress, they are released and translocated to the Golgi, where they are cleaved by S1P and S2P proteases, after which they are released and transported to the nucleus [21,22].

Apart from the membrane-tethered bZIPs, other bZIP TFs have also been shown to be involved in stress responses. For example, in salt stressed roots, Group S bZIP1 and bZIP53 function to alter carbon and amino acid metabolism in such a way to provide the necessary enzymes that utilize carbon skeletons from proteins in order to keep up with energy demands under stress [23]. Moreover, Group A bZIPs have been shown to function in Abscisic acid (ABA) signaling, with their expression induced under conditions that bring about a water deficit (e.g., drought, salinity, and cold; [24]). Once induced, they function to counteract the negative effects of a water deficit. For example, following drought stress, bZIP37 (ABF3) participates in stomatal closure and reprograms metabolism to ensure the accumulation of protective osmolytes, or, it directly induces the expression of late embryogenesis abundant (LEA) genes to protect cells from dehydration [25].

We have previously characterized bZIP18, a Group I bZIP TF with pollen-enriched expression, and demonstrated that its localization is partitioned between the nucleus and cytoplasm [13]. Comparative transcriptomics of *bzip18/−* and Col-0 wild type pollen revealed that bZIP18 most likely functions as a repressor, due to the number of upregulated genes observed in the mutant. In the same study, we identified bZIP52 as an interacting partner of bZIP18. bZIP52 is a close homolog of bZIP18, and its localization has also been shown to be partitioned between the cytoplasm and nucleus [26]. A series of studies on bZIP51 (VIRE2-interacting protein 1, VIP1) and other members of Group I bZIP TFs (among others also bZIP52), have shown their accumulation in the nucleus following mechanical [27] or hypo-osmotic stress [26,28], where it leads to the expression of stress-related genes [29,30]. The finding that its localization is not strictly nuclear suggested a plausible role for bZIP18 in some form of stress response, similar to that of bZIP52. The aim of the current study was to determine whether bZIP18 and bZIP52 localization is affected by heat stress and if so, whether they play a role in the heat stress response.

## 2. Results

### 2.1. bZIP18 and bZIP52 Accumulate in the Nucleus Following Heat Stress

**Figure 1:**
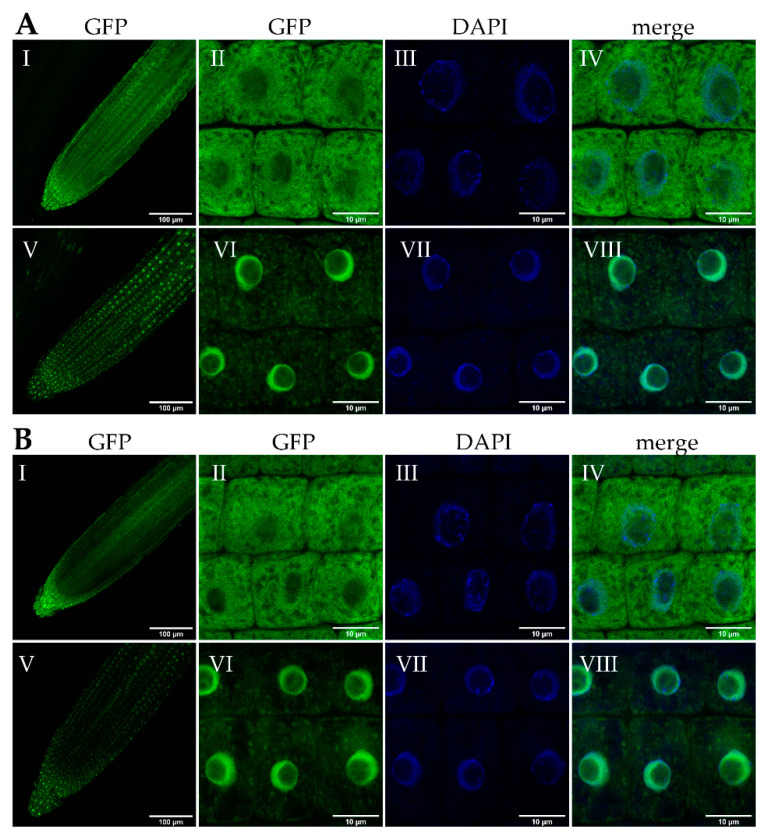
Heat stress brings about nuclear accumulation of bZIP18 and bZIP52. Confocal images of seven-day old seedlings expressing bZIP18-GFP (**A**) (GFP, Green Fluorescent Protein) or bZIP52-GFP (**B**) subjected to heat stress for 1 h. Sections A and B show nuclear and cytoplasmic localization of AtbZIP18-GFP and AtbZIP52-GFP respectively, in the primary root of *A. thaliana* seedlings under control conditions (I–IV) and their re-localization into nuclei following heat stress (V–VIII). Panels I and V show the whole root apex, while the remaining panels (II–IV, VI–WIII) show details of root cells. Nuclear localization was confirmed by DAPI staining (blue fluorescence, Panels VII).

Previous reports of Group I bZIP proteins have shown that several of these TFs are both cytosolic and nuclear in localization; however, they accumulate in nuclei following stress (e.g., mechanical stress, hypo-osmotic stress) [26,27,31]. To determine whether bZIP18 and bZIP52 accumulate in nuclei following heat stress, we subjected transgenic seedlings expressing bZIP18-GFP (Green Fluorescent Protein) or bZIP52-GFP fusion proteins under their native promoters to a heat stress treatment and investigated their localization. Seven days-old seedlings were heat stressed for 1 h at 42 °C and their roots were examined by confocal microscopy. Under control conditions, localization of bZIP18-GFP and bZIP52-GFP proteins was both cytoplasmic and nuclear in the whole root apex and in individual root cells (Figure 1A,B, panels I–IV). Interestingly, both fusion proteins were absent from nucleoli. Following heat stress, we observed prevailing nuclear accumulation for both bZIP18-GFP and bZIP52-GFP, demonstrated through co-localization with 4′,6-Diamidino-2-phenylindole dihydrochloride (DAPI), a fluorescent dye for nucleic acid staining (Figure 1A,B, panels V–VIII). The GFP signal was again excluded from the nucleoli. Furthermore, the nuclei of root cells became more compact after heat stress, compared to the more diffused structure of control nuclei.

### 2.2. bZIP18 and bZIP52 Are Not ER-Sequestered, but Rather Shuttle between the Nucleus and Cytoplasm through Phosphorylation and 14–3–3 Binding

**Figure 2:**
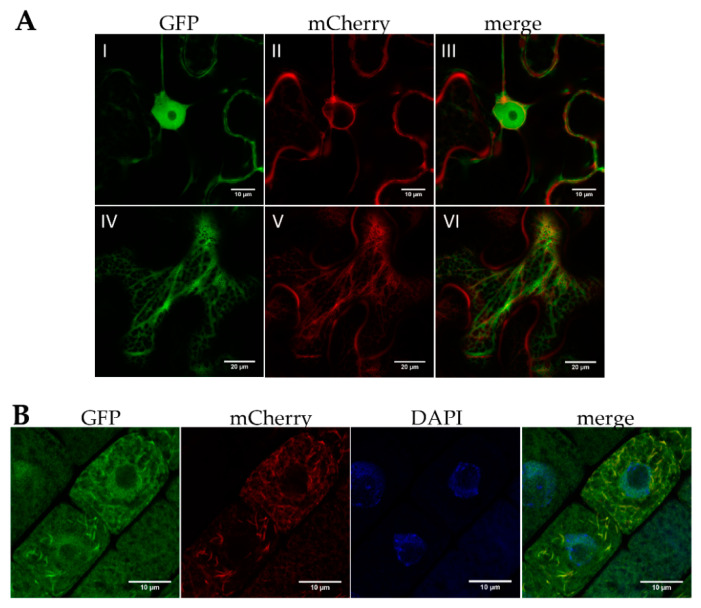
AtbZIP52 only partially co-localizes with the endoplasmic reticulum (ER). (**A**) Confocal images of *N. benthamiana* leaf epidermal cells co-expressing bZIP52-GFP (green fluorescence) and ER marker with mCherry (red fluorescence). Panels I–III show a region with a nucleus, while panels IV–VI show a region containing an ER network. (**B**) Root cells of seven-day old seedlings (*A. thaliana* stable lines) expressing the same proteins and stained with DAPI (blue fluorescence) for visualization of nuclei.

Of the bZIPs implicated in the heat stress response (bZIP17, bZIP28, and bZIP60), some degree of association with the ER is documented [8,32]. These ER-sequestered bZIPs normally have transmembrane domains, a feature that is lacking in bZIP18 and bZIP52 (Figure S1). We have previously shown that bZIP18 partially co-localizes with the ER [13]. Similarly, only partial co-localization was found for bZIP52-GFP co-expressed with the ER marker in *N. benthamiana* leaf epidermal cells (Figure 2A) and in root cells of *A. thaliana* (Figure 2B). A negative control included free GFP co-expressed with the ER marker (Figure S2).

**Figure 3:**
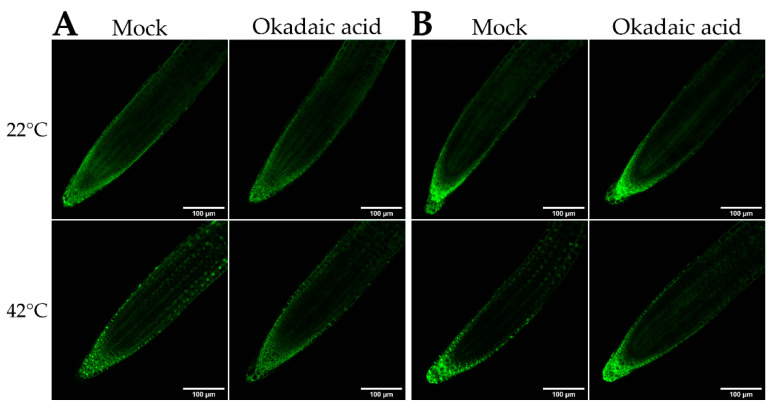
Dephosphorylation is required for bZIP18 and bZIP52 translocation to the nucleus upon heat stress. Five-day old seedlings expressing bZIP18-GFP (**A**) or bZIP52-GFP (**B**) were transferred to liquid ½ MS media without (Mock) or with 5 μM Okadaic Acid for 30 min and subjected to heat stress (42 °C) for another 30 min or left at room temperature (22 °C) and analyzed for GFP signal.

It was reported that under control conditions, VIP1 (Group I bZIP) is cytoplasmic in phosphorylated form, and becomes dephosphorylated following stress, allowing its re-localization to the nucleus [27]. To determine whether a similar mechanism involving dephosphorylation plays a role in bZIP18 and bZIP52 re-localization to the nucleus under heat stress, we subjected 5 days-old transgenic seedlings to an Okadaic Acid (OA) treatment. Okadaic acid acts as an inhibitor of protein phosphatase 2A. Under heat stress conditions, bZIP18-GFP and bZIP52-GFP accumulated in the nuclei of the root apex (Figure 3) when subjected to “mock” treatments (liquid ½ MS media without OA). However, when seedlings were subjected to an OA treatment and heat stress, bZIP18-GFP and bZIP52-GFP nuclear accumulation was reduced (Figure 3). 

**Figure 4:**
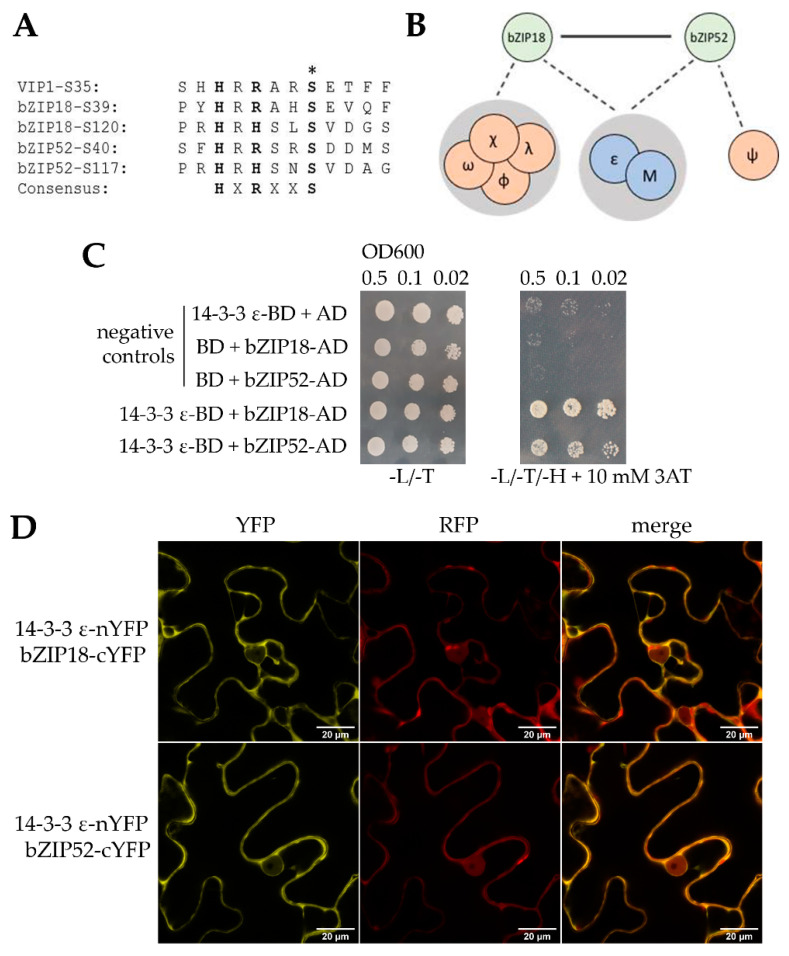
bZIP18 and bZIP52 have two 14–3–3 motifs and interact with 14–3–3 ε. (**A**) Amino acid sequence analysis of bZIP18 and bZIP52 revealed two motifs recognized by 14–3–3 proteins within the HXRXXS motif (bold letters), with serine (indicated by an asterisk) representing the phosphorylatable residue. (**B**) Putative 14–3–3 interacting partners of bZIP18 and bZIP52 identified through pull-down experiments performed on protein extracts of seven-day old seedlings expressing bZIP18-GFP and bZIP52-GFP. (**C**,**D**) bZIP18 and bZIP52 interact with 14–3–3 ε. (**C**) Protein-protein interactions evaluated by yeast two hybrid. Baits were expressed as GAL4 DNA-binding domain (BD) fusions and prey as GAL4 activation domain (AD) fusions. Bait and prey constructs were co-transformed into MAV203, and colonies spotted onto control (−L/−T) and phenotyping (−L/−T/−H + 10 mM 3AT) plates. Plates were incubated at 28 °C for 5 days prior to taking photos. Negative controls are indicated. When bZIP18 and bZIP52 were co-expressed with 14–3–3 ε, yeast cells were able to grow on media lacking histidine, indicating positive interactions. (**D**) Protein-protein interactions evaluated by BiFC assays. nYFP-fused 14–3–3 ε was co-expressed with either cYFP-fused bZIP18 or bZIP52 in *N. benthamiana* epidermal cells, and Yellow Fluorescent protein (YFP) signals detected using confocal microscopy. Reconstitution of the YFP fluorophore indicates a positive interaction, while Red Fluorescent protein (RFP) serves as a transformation control. YFP reconstitution was observed for both bZIP18 and bZIP52 when co-expressed with 14–3–3 ε.

Under control conditions, phosphorylated bZIP TFs are bound by 14–3–3 proteins, which are involved in their sequestration in the cytoplasm [27,28]. Physical interactions between Group I bZIPs and 14–3–3 proteins have been demonstrated for VIP1 and bZIP52 [28]. To determine whether this holds true for bZIP18 also, and to confirm the findings of Tsugama et al. (2019) for bZIP52, we analyzed the respective protein amino acid sequences. We found two motifs in each protein with a candidate serine residue, a putative phosphorylation target recognized by 14–3–3 proteins (Figure 4A). The candidate serine positions in bZIP18 corresponded to S39 and S120, while in bZIP52 they corresponded to S40 and S117. To determine whether our bZIPs bind to 14–3–3 proteins, we isolated proteins from 7-day old seedlings over-expressing bZIP18-GFP or bZIP52-GFP and used the isolated proteins for pull-down assays using GFP-Trap^®^ beads, where potential interacting partners of both were identified by LC-MS/MS. We have identified several 14–3–3 proteins that putatively interact with either one or both of the bZIP proteins (Figure 4B; Table S1). Of them, both bZIP18 and bZIP52 pulled down 14–3–3 ε (GRF10). To verify their interaction, we carried out both yeast two hybrid (Y2H) and bimolecular fluorescence complementation (BiFC) analyses. 

We have previously found that full length sequences of bZIP genes fused to the DNA binding domain (BD) of GAL4 in the Y2H system brought about auto-activation in the absence of a prey plasmid carrying the activation domain (AD; [13]). To this effect, we cloned full length sequences of the two bZIP genes into the prey plasmid as AD fusions and 14–3–3 ε into the bait plasmid as a BD fusion. We observed growth for the colonies co-expressing bZIP18/bZIP52 and 14–3–3 ε, confirming that they indeed interacted (Figure 4C). As an independent confirmation step, we carried out BiFC in *N. benthamiana* leaf epidermal cells co-expressing bZIP18-cYFP/bZIP52-cYFP and 14–3–3 ε-nYFP. We saw reconstitution of YFP for bZIP18 and bZIP52 when co-expressed with 14–3–3 ε, confirming their interaction (Figure 4D). Moreover, the YFP signal localized only to the cytoplasm, compared to the YFP signal which originated from both the nucleus and cytoplasm when bZIP18 and bZIP52 were co-expressed (Figure S4). 

**Figure 5:**
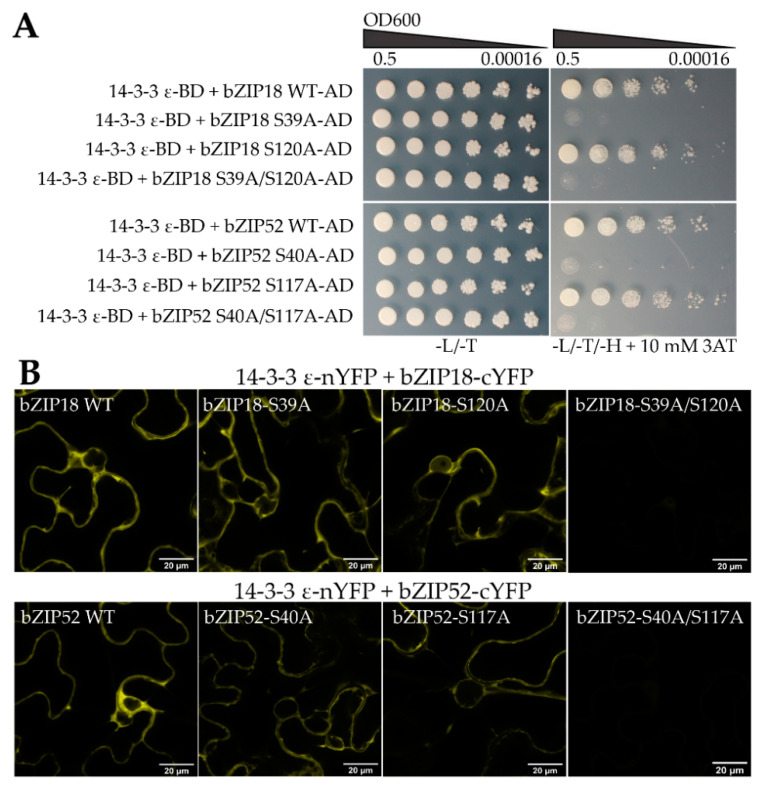
Mutational analyses reveal which residues are important for 14–3–3 binding. (**A**) Yeast two hybrid assays with variants of bZIP18 and bZIP52. Bait (14–3–3 ε-BD) and prey (bZIP-AD) plasmids were co-transformed into the MaV203 and colonies spotted onto control (−L/−T) and phenotyping (−L/−T/−H + 10 mM 3AT) plates. Plates were incubated at 28 °C for 5 days. Mutation of the first residue or both simultaneously resulted in yeast cells unable to grow on media lacking histidine, indicating no interaction. (**B**) BiFC analyses of cYFP-fused variants of bZIP18 and bZIP52 against nYFP-fused 14–3–3 ε in *N. benthamiana* epidermal cell revealed YFP signals (positive interaction) for the single mutations, but not the double.

**Figure 6:**
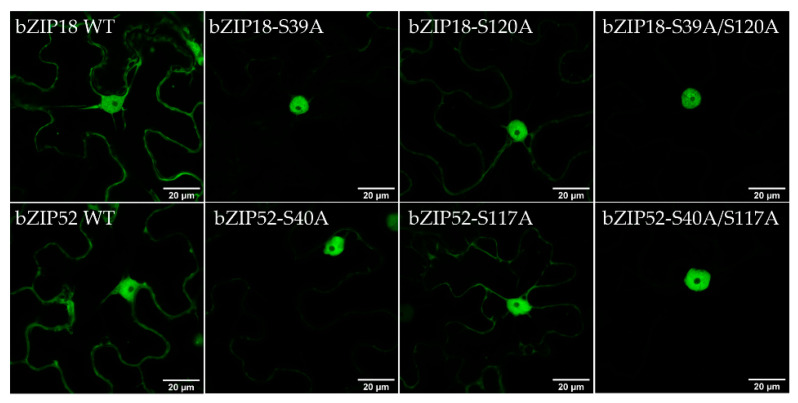
Mutation of both serine residues simultaneously leads to strict nuclear localization. Confocal images of *N. benthamiana* expressing bZIP-GFP variants of bZIP18 and bZIP52. Localization in the cytoplasm is pronounced in WT expressing plants, compared to plants expressing single mutant variants of each bZIP. Only when both residues are mutated (bZIP18-S39A/S120A and bZIP52-S40A/S117A) is strict nuclear localization observed for both TFs.

To determine whether both serine residues are important for 14–3–3 binding, we carried out mutational analyses by replacing either one or both residues with alanine (A), and investigating how their interaction with 14–3–3 ε is affected. Y2H analyses demonstrated that when the first residue (bZIP18-S39A, bZIP52-S40A) was replaced by alanine, yeast cells were unable to grow on media lacking histidine, indicating that no interaction took place (Figure 5A). However, when the second residue (bZIP18-S120A, bZIP52-S117A) was replaced, they were able to grow, implying interaction. When both residues were replaced by alanine at the same time, no growth was observed on media lacking histidine. BiFC analyses, performed in parallel, gave only partially complementary results. Both single-amino acid mutants showed a weak YFP signal. It was only the double mutant where we observed no YFP signal indicating that only in the double-amino acid mutant no YFP reconstitution happened (Figure 5B and Figure S3). To determine whether 14–3–3 binding does in fact cause bZIP sequestration to the cytoplasm, we investigated how their localization was affected by mutation of these serine residues. Wild type and mutant variant GFP fusions were transiently expressed in *N. benthamiana* epidermal cells and their localization was investigated using confocal microscopy (Figure 6). bZIP18-S39A showed a strong nuclear signal with a very weak signal remaining in the cytoplasm. bZIP18-S120A also had a strong signal in the nucleus with a weaker signal from the cytoplasm compared to WT, but stronger than in bZIP18-S39A. The double mutant, bZIP18-S39A/S120A, however, showed a strict nuclear localization. Similar results were obtained for bZIP52. Both bZIP52 single-amino acid mutations showed strong nuclear and weak cytoplasmic signals, weaker for the more proximal mutated serine. The weakest cytoplasmic signal was then detected for the double-amino acid mutations. Taken together, both bZIP18-S39A/S120A and bZIP52-S40A/S117A constructs drive a strict nuclear GFP localization pattern. 

### 2.3. Differential Gene Expression Highlights lncRNA-Mediated Regulation

**Figure 7:**
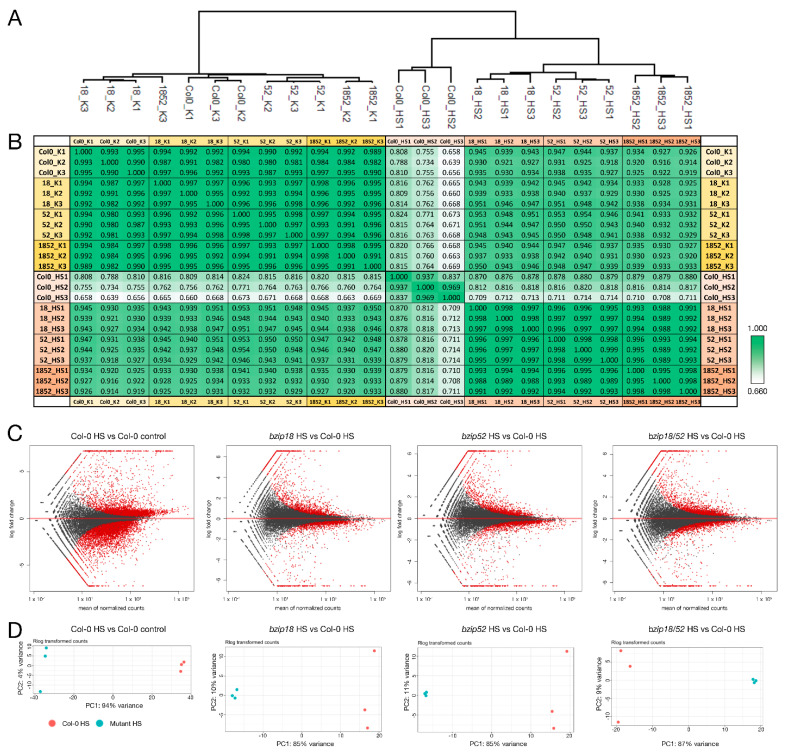
Analyses of heat-stressed (HS) seedling transcriptomes. (**A**) Hierarchical clustering of regulated log-transformed read counts of four genotypes-Col-0 wt, and homozygous *bzip18*, *bzip52*, *bzip18/bzip52* mutants based on Pearson correlation distance shows three distinctive clusters. (**B**) Correlation between individual datasets. Each cell contains Pearson’s correlation coefficient for the respective samples. (**C**) MA plot of gene expression in HS homozygous *bzip18*, *bzip52*, *bzip18/bzip52* mutants compared to Col-0 wt. As a control, heat stressed (HS) Col-0 seedlings were compared to non-stressed (control) ones. Genes with adjusted *p*-value < 0.05 are shown in red. (**D**) Principal Component Analysis (PCA) based clustering of regularized log-transformed read counts of the same sample combinations as in (**C**).

To further explore the possible involvement of bZIP18 and bZIP52 in the HSR, we aimed to demonstrate the sets of transcripts associated with the stress response in wild type and *bzip* mutant plants. For the analyses, one-week-old seedlings were used both heat-stressed (42 °C for 1 h) or grown under control conditions. We compared wild type Col-0 plants with those harboring homozygous mutations in *bzip18, bzip52* and double homozygous mutation in *bzip18/52* genes. To this effect, we applied RNA-seq and performed comparative transcriptomic analysis. For each sample, three biological replicates were used. Quality of RNA and total number of reads per biological replicate are summarized in Table S2. Normalized expression values are presented in Table S3. The application of hierarchical clustering (Figure 7A) to visualize the relationship among the individual samples and correlation evaluation of all datasets (Figure 7B) demonstrated the close relationship of the corresponding samples. All these analyses also unequivocally showed the consistency among the replicates. Moreover, the obtained normalized expression matrices were confirmed as being of good quality for downstream analyses.

Under standard conditions, there were no massive differences between Col-0 and mutant plants (Figure 7A,B). These differences became more apparent after the application of heat stress. In this situation, wild type plants responded differently from the mutants. Therefore, we compared the transcriptomes of wt and mutant seedlings only in the HS conditions. This allowed us to analyze only one parameter at a time. MA plot of gene expression in *bzip* mutants compared to Col-0 WT showed the extent of differential gene expression in response to heat stress (Figure 7C). It showed that the effect of the mutations was lower than that of the HS. Moreover, both single mutants responded similarly as the double mutant (Figure 7C). It was confirmed also by the principal component analysis (PCA) that always grouped replicates of the same developmental stage (Figure 7D).

**Figure 8:**
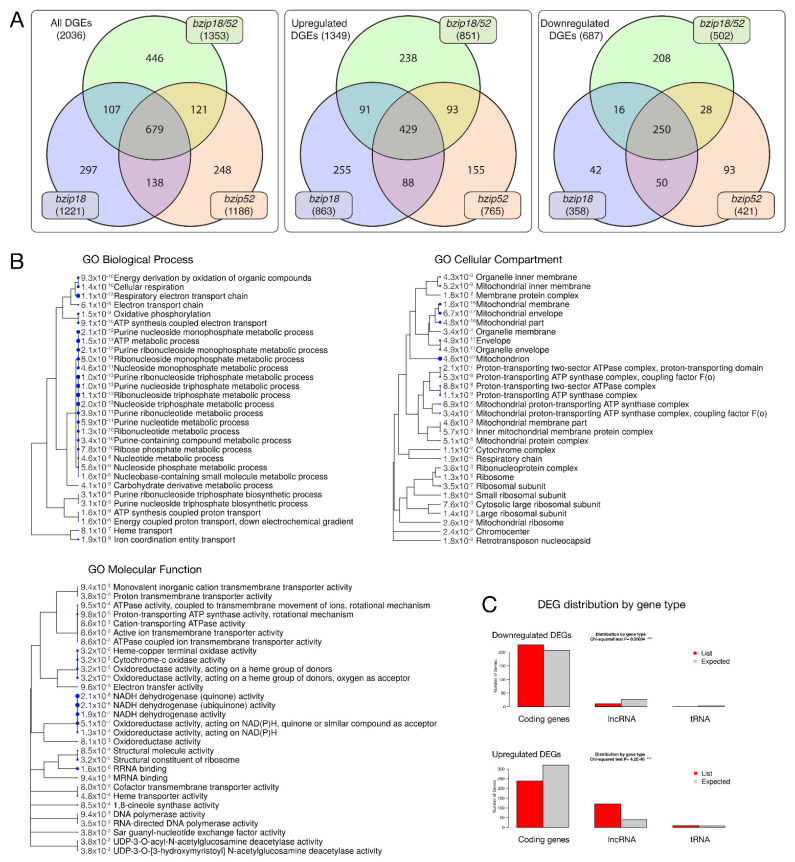
Differential gene expression in seedling transcriptomes. (**A**) Venn diagrams showing the number of unique and overlapping differentially expressed genes (DEG). All DEGs are compared with DEGs upregulated and downregulated in mutants. (**B**) Gene Ontology (GO) summary of 250 DEGs downregulated in all three mutant genotypes. Blue dots reveal the relevance of GO categories (**C**) DEG distribution by gene type, coding genes, lncRNAs, and tRNAs; observed (List) and expected values are compared separately for DEGs downregulated and upregulated in HS mutant seedlings.

Categorization of differentially expressed genes (DEGs) in both *bzip18/−* and *bzip52/−* single mutants and the homozygous *bzip18/bzip52* double mutant revealed the high proportion of DEGs shared by all three mutant genotypes (Figure 8A). It was almost exactly one third of genes differentially expressed in at least one genotype (679/2,036). Within individual genotypes, this share was higher, always over 50% (55% in *bzip18*, 57% in *bzip52* and 50% in *bzip18/52*). In all genotypes, there were more genes upregulated than downregulated in mutants. Interestingly, Gene ontology (GO) categorization of the genes differentially expressed in all three mutant genotypes (679 genes) did not identify any overrepresented category among the 429 upregulated genes. On the contrary, several GO categories were over-represented among 250 genes downregulated in all three mutant genotypes. These comprised predominantly genes involved in energy metabolism, namely ATP metabolism, cellular respiration and oxidative phosphorylation, stress response, and translation (Figure 8B). Accordingly, the most overrepresented cellular components were various mitochondrial compartments and ribosomes, both ribosomal subunits.

Interestingly, although the upregulated genes are quite diverse, not showing any shared GO category, they were clearly enriched with long non-coding RNAs (120 lncRNAs). This enrichment was particularly apparent when compared to that of downregulated genes (Figure 8C). 

### 2.4. Genome-Wide Discovery of Regions Bound by bZIP18 and bZIP52 Reveal That They Target the Same Set of Genes

To identify genomic regions targeted by the bZIP18 and bZIP52 proteins, we performed chromatin immunoprecipitation and sequencing (ChIP-seq) analyses on transgenic lines over-expressing (OX) bZIP-GFP fusion proteins under CamV-35S promoters (pGWB5::bZIP-GFP). This was done for single mutant lines (bZIP18-GFP and bZIP52-GFP) and a double mutant line (bZIP18-GFPxbZIP52-GFP), which was obtained by conventional crossing of the single OX lines. With regards to the double mutant line, we have previously shown that bZIP18 and bZIP52 interact via a yeast two hybrid assay [13], and this result was independently confirmed by BiFC (Figure 4). We used a line expressing free GFP as a negative control. Three-week-old seedlings were used for ChIP-seq performed in three biological replicates per sample. Summarizing sequencing statistics are shown in Table S5. 

**Figure 9:**
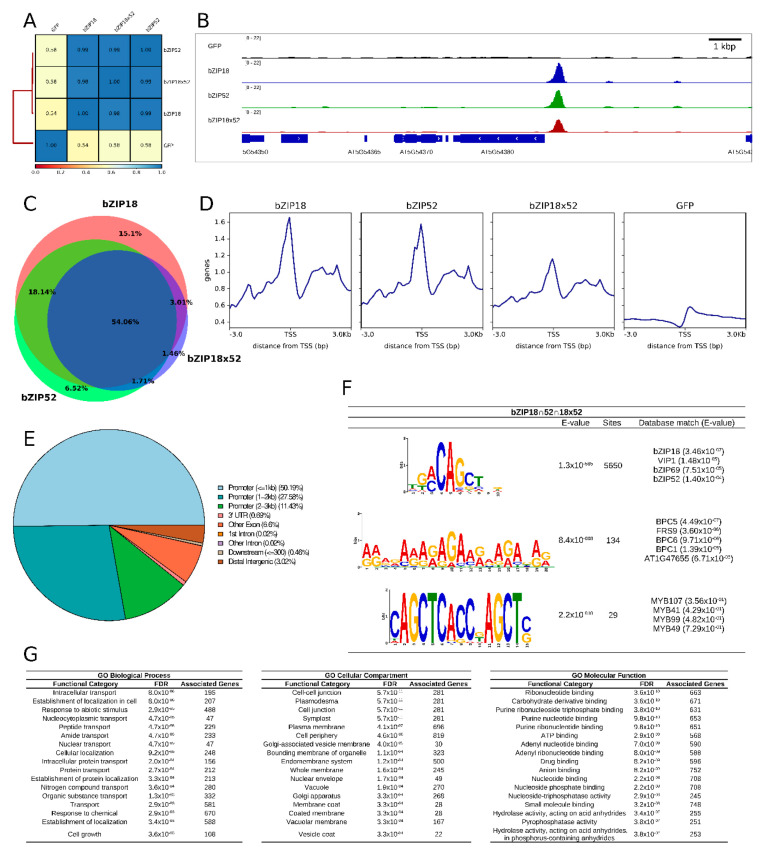
ChIP-seq analyses of plants over-expressing bZIP18, bZIP52, and bZIP18x52. (**A**) Pearson correlation matrix showing similarity between ChIP-seq samples. The scores were calculated based on mapped read coverage data. (**B**) Integrated Genome Viewer (IGV) snapshots with an example of loci enriched in the ChIP-seq data. (**C**) Venn diagram showing overlap between genes associated with regions enriched either for bZIP18 (red), bZIP52 (green), or bZIP18x52 (blue). (**D**) Read coverage at transcription start sites (TSS) and adjacent regions of genes associated with enriched regions, only genes common to all three bZIP lines are shown. (**E**) Percentage of enriched regions common to all three bZIP lines that overlap given genomic element. (**F**) The three most abundant motifs discovered at regions enriched in all three bZIP lines (total number of regions analyzed: 5670). The last column shows top five matches in the ArabidopsisDAPv1 database (O’Malley et al. 2016). (**G**) The 20 most significant Gene Ontology (GO) terms that were found among genes common to all three bZIP lines (5069 genes were subjected to the analysis).

The coverage comparison after read mapping by Pearson correlation showed high similarity between the samples (Figure 9A and Figure S5). Based on the enrichment of mapped reads in the peaks, we calculated the number of bound regions (with FDR = 0.05), and found 10,331, 9095, and 6586 regions bound by bZIP18, bZIP52, and bZIP18x52 respectively. We subsequently identified the genes associated with these regions, which are likely regulated by these bZIP TFs (Figure 9B, Table S6). An example of such a gene is shown in Figure 8B. Overlap between the genes identified in individual samples showed very high similarity between the targets of both bZIP TFs (Figure 9C). Interestingly, the bZIP18x52-associated genes formed mostly a subset of the genes associated with bZIP18 and bZIP52, a consequence of generally lower signal in the bZIP18xbZIP52 sample (Figures S6 and S7). Given the large overlap between the samples, we focused mainly on shared regions for further analyses, which included 5069 genes. Looking at the read coverage over the transcription start sites (TSS) for this subset of genes, we found a clear peak at the TSS region (Figure 9D). This peak was already apparent from the read coverage over TSSs of all genes in Arabidopsis genome (Figure S8). Further analyses showed that 89% of the peaks are located in promoter regions covering 3 kbp upstream from TSS, or 50% when considering only 1 kbp upstream from TSS. Besides promoters, a significantly lower number of peaks were identified in exons (6.6%) or in intergenic regions (3%). All of the analyses gave almost identical results when done for each bZIP-overexpressing sample separately (Figure 9E, Figures S9 and S10).

Transcription factors bind to target sites based on their specific DNA sequences [33]. We identified one especially prominent motif, TGMCAGCTND, in the DNA sequences bound by the analyzed bZIP TFs (Figure 9F). Moreover, the MEME tool identified this motif in 5650 (99%) of input sequences (*N* = 5670). Comparison of this motif with a database of known motifs [34], revealed high similarity to motifs bound by bZIP18, VIP1 (bZIP51), bZIP69, and bZIP52. The other identified motifs were found in only a small fraction of the targeted regions (2% and less), and were similar to a different set of TFs (Figure 9F and Figure S11, Supplementary File S1). To gain better insight into the set of genes targeted by these bZIP TFs, we performed the gene ontology (GO) categorization of the associated genes (Figure 9G, Table S7). GO data revealed that many enriched genes were involved in transport and localization (intracellular transport, nuclear transport, cellular localization, etc.), while other genes were involved in responses to stimuli. In keeping with this, many protein products of these genes localized to plasmodesmata, or to other membrane compartments. 

**Figure 10:**
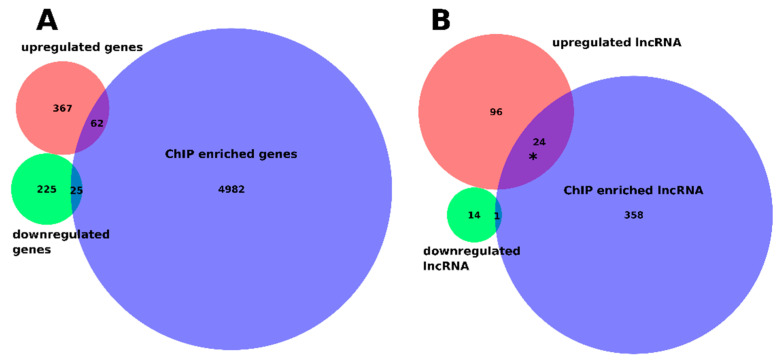
Comparison of genes differentially expressed in mutants under heat stress conditions with genes identified by ChIP-seq. (**A**) Venn diagram showing overlap between genes associated with regions commonly enriched for bZIP18, bZIP52, or bZIP18x52 (blue) with genes upregulated (red) or downregulated (green) in bzip18, bzip52, and bzip18/52 heat stressed mutants. (**B**) Same as in (**A**) but only for lncRNAs. Asterisk denotes significant overlap (calculated by hypergeometric distribution function).

Next, we studied the relationship between the ChIP-seq and RNA-seq data. Due to the small number of DEGs in the control dataset (Figure S12), we focused on DEGs in the HS conditions. Overlap between genes upregulated and downregulated in all three mutants with genes that showed enrichment for the ChIP-seq signal in all three lines was 14% and 10% of the DEGs, respectively (Figure 10A). This also did not show significant results in the GO analysis. Because the previous results showed possible involvement of lncRNAs in the bZIP-regulated heat stress response, we looked at these separately (Figure 10B). Within the lncRNA set, the overlap with upregulated genes was 20% (higher than expected by chance, *p* = 0.002), and one gene was common with the small set of downregulated lncRNA genes (6.7%).

## 3. Discussion

We have previously characterized bZIP18, a Group I bZIP TF, and demonstrated its involvement in pollen development [13]. In the same study, we revealed that it dimerizes with another Group I bZIP TF, bZIP52. Both of these TFs are close homologs of VIP1 (bZIP51), which has been shown to accumulate in the nucleus following mechanical or hypo-osmotic stress [26,27,28]. We were interested in whether bZIP18 and bZIP52 respond to heat stress, and if so, whether they are implicated in the heat stress response.

As a starting point we investigated how the localization of bZIP18 and bZIP52 are affected by heat stress (Figure 1). Under control conditions, their localization is in agreement with previously published data for Group I bZIPs, which have demonstrated that under non-stressed conditions, these proteins are partitioned between the cytoplasm and nucleus [13,26,28]. Following heat stress however, strong nuclear accumulation can be observed for both TFs. This re-localization to the nuclei called for investigation into how these proteins move between the cytoplasm and nucleus. We were able to rule out ER-sequestration, as neither bZIP18 nor bZIP52 possess transmembrane domains (Figure S1) or did not fully co-localize with the ER (Figure 2). The most likely mode of action for bZIP18 and bZIP52 re-localization to the nucleus probably follows that of their close homolog VIP1 (bZIP51). The latter requires TF dephosphorylation to facilitate its accumulation in the nucleus [27,28]. An Okadaic Acid (OA; phosphatase inhibitor) treatment of seedlings prior to HS demonstrated that bZIP18 and bZIP52 require dephosphorylation to move to the nucleus (Figure 3). Moreover, our results indicate that heat stress brings about dephosphorylation of bZIP18 and bZIP52. Finally, they are most likely dephosphorylated by Protein Phosphatase 2A (PP2A), which is specifically inhibited by OA. 

In line with this, VIP1 possesses serine residues that are phosphorylated, and in phosphorylated form, are bound to 14–3–3 proteins, which sequester them to the cytosol [27,28]. Through multiple sequence alignment with VIP1, we were able to identify two candidate serine residues within HXRXXS motifs bound by 14–3–3 proteins in both bZIP18 and bZIP52 (Figure 4A). These included S39 and S120 in bZIP18 and S40 and S117 in bZIP52. bZIP52 was shown previously to interact with 14–3–3 κ and 14–3–3 λ; however, no such data exists for bZIP18. Pull-down assays revealed 14–3–3 ε as a putative interacting partner of both bZIP18 and bZIP52 (Figure 4B), with Y2H and BiFC experiments confirming that they do interact (Figure 4C,D). Moreover, BiFC experiments added confidence to the hypothesis that 14–3–3 proteins sequester bZIP TFs to the cytoplasm. When looking at the YFP localization in Figure 4D, one can see that the signal is coming predominantly from the cytoplasm, with negligible signal coming from the nucleus. When BiFC was used to test the interaction between bZIP18 and bZIP52, the signal from the nucleus was very strong compared to the cytoplasm (Figure S4A).

It has been found that mutation of some of these serine residues disrupts bZIP binding to 14–3–3 proteins. For example, mutation of two of the three VIP1 residues (S35A and S115A) in *A. thaliana* disrupted 14–3–3 λ and 14–3–3 κ binding [28]. In the same study, bZIP52 was also subjected to mutational analysis. There they mutated two serine residues in two motifs (S38A and S40A in the first motif, and S115A and S117A in the second motif). Mutation of the S38A and S40A in the first motif disrupted 14–3–3 λ and 14–3–3 κ binding. In our study, we focused solely on S40 and S117 in bZIP52, which we believed to be the true phosphorylatable serine residues contained within these motifs. Considering the position of S38 and S115 within the HXRXXS motif, neither occupies the correct position. S38 and S115 occupy a serine residue immediately following arginine (R), even though the residue prone to phosphorylation resides two positions upstream of R (HXRXXpS) [27,35]. Mutational analyses of bZIP18 and bZIP52 revealed discrepancies between Y2H and BiFC results. Y2H analyses showed that mutation of the first serine residue (bZIP18-S39A and bZIP52-S40A) abolishes 14–3–3 ε binding (Figure 5A); however, this was not the case for BiFC (Figure 5B). Only when both residues were mutated at the same time was 14–3–3 ε binding abolished. Discrepancies among Y2H and BiFC interactions have been reported before [36]. BiFC is known to give more false positives than other interaction analyses [37], however, if we take into consideration the localization results (Figure 6), we can give more confidence to the BiFC results. Localization data show that disruption of one residue at a time is not enough to obliterate bZIP localization in the cytoplasm. Only when both residues are mutated do we see a strict nuclear localization pattern. The findings for bZIP52 are in agreement with Tsugama et al., (2019) [28] with regards to both Y2H and localization data. In the Y2H system, no growth was observed in the bZIP52-S38A/S40A variant; however, when expressed in lettuce cells as a GFP fusion protein, localization was still observed in the cytoplasm, illustrating that Y2H brings about a false negative result in this case. Taken together, the BiFC and localization data suggest that both motifs/residues appear to be important for 14–3–3 ε binding.

ChIP-seq data revealed that bZIP18 and bZIP52 target the same set of genes, with 99% of target genes shared by the two TFs containing the CAGCT motif in their promoters (Figure 9). This motif is also bound by VIP1 and bZIP29 [30,38], two Group I bZIPs. GO analysis revealed a diverse set of target genes in multiple categories regulated by bZIP18 and bZIP52, pointing to pleiotropic roles for these TFs in seedlings. Moreover, comparative transcriptomic analysis of *bzip*-deficient mutant lines further confirmed the coordinated action of both bZIP18 and bZIP52 TFs in response to heat stress. Three genotypes were compared, *bzip18/−* and *bzip52/−* single mutants and the homozygous *bzip18/52* double mutant. Of genes differentially expressed in response to heat stress treatment in each genotype, 50–57% was shared by all three genotypes. The examined bZIP TFs are likely to act predominantly as repressors since 63–70% of DEGs were upregulated in individual mutant genotypes and, accordingly, 30–37% DGEs were downregulated. Although underrepresented, downregulated DEGs formed a more specific category comprising predominantly genes involved in energy metabolism, namely ATP metabolism, cellular respiration and oxidative phosphorylation, stress response, and translation. On the contrary, upregulated DEGs did not comprise any significantly overrepresented GO category; 28% of them encoded long non-coding RNAs. lncRNAs were previously shown to regulate gene expression in stress response in several species including *Triticum aestivum* [39], *Brassica rapa, ssp. chinensis* [40], *Cucumis sativus* [41], and *Brassica juncea* [42], however, the regulation of lncRNA expression by bZIP TFs under heat stress has not been reported yet. On the contrary, bZIP TFs were already reported to be targets of stress-regulated lncRNAs in *Pistacia vera* [43] and in *Cleistogenes songorica* [44]. Finaly, the lack of consistency among upregulated genes and a reasonable coherence among the downregulated genes, together with the upregulation of numerous lncRNAs suggests the possible mechanism of the upregulation of the downstream stress-responsive genes via lncRNAs alongside their direct transcription regulation.

## 4. Materials and Methods 

### 4.1. DNA Constructs

To create stable *Arabidopsis thaliana* lines expressing bZIP-GFP fusion proteins, we generated plasmids harboring the complete genomic sequence of each *bZIP* gene (including their native promotors without stop codons) C-terminally fused to GFP. *AtbZIP18* (At2g40620) [13] and *AtbZIP52* (At1g06850) were PCR-amplified from *A. thaliana* leaf genomic DNA using gene specific primers (Table S8) and Phusion^TM^ High-Fidelity DNA Polymerase (Thermo Fisher Scientific, Waltham, MA, USA). Amplicons were cloned into the pENTR^TM^/D-TOPO entry vector (Thermo Fisher Scientific, Waltham, MA, USA) and consequently recombined into the pB7FWG,0 expression vector harboring the eGFP marker using LR Clonase^TM^ (Thermo Fisher Scientific, Waltham, MA, USA) ([45]; https://gatewayvectors.vib.be/). 

For transient expression in *N. benthamiana* epidermal cells and the generation of stable over-expression lines, we used the plasmid described in [13] for AtbZIP18. To generate the construct for AtbZIP52, its coding sequence was PCR-amplified from *A. thaliana* inflorescence cDNA using gene specific primers (Table S8) and cloned into the pENTR^TM^/D-TOPO entry vector (Thermo Fisher Scientific, Waltham, MA, USA). A sequenced entry clone was subsequently used in a LR Clonase^TM^ reaction to create the pGWB5 (C-terminal GFP fusion under the 35S promoter) expression vector [46]. 

For BiFC experiments, *AtbZIP18*, *AtbZIP52*, *At14–3–3 ε* (At1g22300), and *AtATA20* (At3g15400) were PCR-amplified from *A. thaliana* inflorescence cDNA by two step PCR using primers specified in Table S8 and Phusion^TM^ High-Fidelity DNA Polymerase (Thermo Fisher Scientific, Waltham, MA, USA). Amplicons were cloned into Gateway pDONR221 entry vectors (Thermo Fisher Scientific, Waltham, MA, USA) carrying either attP1-P4 or attP3-P2 recombination sites using the BP Clonase^TM^ II enzyme mix (Thermo Fisher Scientific, Waltham, MA, USA). Entry clones were subsequently used in LR Clonase^TM^ II Plus (Thermo Fisher Scientific, Waltham, MA, USA) reactions to create pBiFCt-2in1-CC [47] expression constructs harboring two protein coding regions C-terminally fused to either the N- or C-terminal part of YFP (e.g., bZIP52-nYFP and bZIP18-cYFP). Verified constructs were used for transient expression in *N. benthamiana* leaves.

For yeast two-hybrid assays (Y2H), the coding sequence of *At14–3–3 ε* was PCR amplified from inflorescence cDNA using Phusion^TM^ High-Fidelity DNA Polymerase (Thermo Fisher Scientific, Waltham, MA, USA) and the primers listed in Table S8. Amplicons were cloned into Gateway pDONR221 (Thermo Fisher Scientific, Waltham, MA, USA), and the entry clones used in LR Clonase^TM^ reactions to create pDEST32 and pDEST22 (Thermo Fisher Scientific, Waltham, MA, USA) bait and prey plasmids respectively. The Y2H plasmids harboring bZIP18 and bZIP52 were generated previously [13].

For CRISPR/Cas9, we utilized the pHEE401E vector harboring the EC1 (egg cell 1) promoter [48] and designed two gRNAs targeted to exon 1 of each *bZIP* gene using specific primer sets (Table S8, *bZIP* target sequences are underlined). The expression constructs were subsequently used for *A. thaliana* transformation.

### 4.2. Mutagenesis

Potential phosphorylation sites in *AtbZIP52* and *AtbZIP18* were mutated using the QuikChange XL Site-Directed Mutagenesis kit (Agilent Technologies, Santa Clarita, CA, United States) following the manufacturer’s instructions. Briefly, pDONR221 entry clones harboring the coding sequences of *bZIP52* and *bZIP18* were amplified with two complementary synthetic oligonucleotide primers (Table S8) containing point mutations that resulted in S40A or S117A substitutions in bZIP52 and S39A or S120A substitutions in bZIP18 using the PfuTurbo DNA polymerase. Parental methylated plasmids were digested with *Dpn*I endonuclease and the mutation containing synthesized DNA was transformed into XL10-Gold Ultracompetent cells. Resulting plasmids were sequenced and correct clones used in LR Clonase^TM^ reactions to create pGWB5, pDEST22, or pBiFC-2in1-CC expression vectors.

### 4.3. Transient Heterologous Expression in Nicotiana benthamiana

*Agrobacterium tumefaciens* competent cells (strain GV3101) were transformed with selected expression clones and selected on YEB medium supplemented with gentamycin (50 µg/mL), rifampicin (50 µg/mL), and a vector specific selection agent (pBiFCt-2in1-CC: spectinomycin 100 µg/mL, pBIN ER-rk: kanamycin 50 µg/mL, pGWB5: kanamycin 50 µg/mL) at 28 °C for 48 h. Colonies were inoculated in the same media lacking agar and grown overnight at 28 °C. Bacterial cells of overnight cultures were pelleted by centrifugation (5 min at 1620 *g*), washed twice, re-suspended, and diluted to an OD_600_ of 0.5 with infiltration medium (10 mM MES pH 5.6, 10 mM MgCl_2_ and 200 µM acetosyringone). A suspension of cells harboring the p19 repressor plasmid was added in a 1:1 ratio with other suspensions to suppress gene silencing and to enhance transient expression [49]. Mixed suspensions were incubated with moderate shaking for 3 h at room temperature and subsequently injected into the abaxial side of 4-week-old *N. benthamiana* leaves. Two to three days after infiltration, tobacco epidermal cells were analyzed microscopically.

### 4.4. Generation of Arabidopsis thaliana Stable Transgenic Lines

Expression clones were transformed into *A. tumefaciens* (strain GV3101) as before, and cultures were used for floral dipping of *A. thaliana* Col-0 wild type plants [50]. Transformants were selected on ½MS medium (0,22% Murashige and Skoog basal medium, 1% sucrose, 0,01% myo-inositol, 0,05% 2-(N morpholino) ethanesulfonic acid, 0,8% agar, pH 5.7 with KOH) supplemented with vitamins (0,01% Niacin, Thiamin and Pyridoxine) and appropriate antibiotics (pB7FWG,0: Basta/Glufosinate ammonium 15 µg/mL, pGWB5: kanamycin 50 µg/mL and hygromycin 25 µg/mL, pHEE401E: hygromycin 25 µg/mL). Plants were cultivated at 22 °C under a 16 h light/8 h dark regime. 

To obtain homozygous plants harboring both pGWB5 constructs, the respective single lines were crossed and homozygous lines selected. For co-localization experiments, homozygous pB7FWG,0 lines were transformed with an endoplasmic reticulum (ER) marker (details in Section 4.6), and homozygous lines were selected. 

For the identification of *A. thaliana* CRISPR *bzip* mutants, particular *bZIP* gene fragments were PCR amplified, purified by QIAquick PCR purification kit (Qiagen, Hilden, Germnay), and sequenced by Sanger sequencing (Eurofins Genomics GmbH, Ebersberg, Germany) using gene specific primers (Table S8). The following independent single and double *bzip* mutant lines were selected: a *bzip18* single mutant with a 242 nt deletion in exon 1 of the *bZIP18* gene, which resulted in changes of amino acid composition from amino acid (AA) at position 34, creating STOP codons at AA positions 37, 41, 50, 52, 58, 59, 62, 69, and 72 in exon 1; a *bzip52* single mutant with homozygous 1 nt addition in both targets of *bZIP52* exon 1 (+A/+A in target 1 and +T/+T in target 2), which resulted in changes of AA composition, resulting in STOP codons at AA positions 25, 78, 82, 101, and 119 in exon 1. For the *bzip1852* double mutant, the same homozygous mutations as *bzip18* and *bzip52* single mutants were selected.

### 4.5. Microscopy

All microscopy images were acquired using the Zeiss LSM880 (Axio Observer Z1, inverted) laser scanning microscope with Definite Focus 2 and the Airyscan detector (excitation 488 nm for GFP/YFP, 561 nm for RFP/mCherry, 405 nm for DAPI). Images were processed using Fiji/ImageJ [51] and Adobe Photoshop CS6 (Adobe, San Jose, CA, USA) softwares. 

### 4.6. ER Co-Localization

Co-localization of bZIP-GFP fusion proteins with the endoplasmic reticulum (ER) marker fused to mCherry, a red fluorescent protein (ER-rk; HDEL signaling sequence; [52]), were investigated in stable lines as well as *N. benthamiana* epidermal cells transiently expressing the respective proteins. Stable lines represented *A. thaliana* transgenic lines expressing bZIP-GFP under their native promotors (pB7FWG,0) transformed with the aforementioned ER marker. For transient expression, overexpression constructs (pGWB5) were introduced into *N. benthamiana* leaf epidermal cells alongside the ER marker. 

### 4.7. Heat Stress Treatment

Seeds of homozygous bZIP lines were sown on ½ MS medium without selection and grown at 22 °C under a 16 h light/8 h dark regime in either a vertical (pB7WG,0) or horizontal arrangement (pGWB5, Crispr bZIP lines). Seven-day-old seedlings were subjected to heat stress treatment. Half of the plates with seedlings were transferred into a growth chamber set to 42 °C for 1h in the light (heat stress variant), half of the seedlings served as a non-stressed control. After the heat stress treatment, the seedlings were put back to 22 °C for 30 min to recover and quickly harvested into liquid nitrogen for further analysis (RNA extraction, pull down). Seedlings for microscopic studies started to be analyzed immediately after heat stress. DAPI staining solution (8 μl of DAPI stock solution in 10 mL of extraction buffer, modified according to [53]) was used for cell nuclei visualization in the heat stress re-localization study. 

### 4.8. Okadaic Acid Treatment

Seeds of homozygous bZIP lines (pB7FWG,0) were sown on solid ½ MS medium (see above Section 4.4) without selection and grown vertically at 22 °C under a 16 h light/8 h dark regime. Five day old seedlings were gently transferred to a solution (liquid ½ MS medium) with or without 5 µM Okadaic Acid (Calbiochem, San Diego, CA, USA) (modified according to [28]) and preincubated for 30 min at 22 °C to allow inhibitor to get inside the root tissues. After preincubation, seedlings were heat stressed at 42 °C for 30 min, which provides sufficient time for the relocalization of GFP signal into the nuclei in the mock treatment. After the heat stress treatment, the seedlings were immediately analyzed for GFP signal using confocal microscopy.

### 4.9. Pull-Down Assays

Seven days-old seedlings expressing bZIP18-GFP and bZIP52-GFP driven by their native promoters (pB7FWG,0) were harvested from petri dishes and flash frozen in liquid nitrogen. Seedlings were ground to a fine powder in a pre-chilled mortar and pestle and 100 mg of material transferred to a 1.5 mL eppendorf tube. One ml of extraction buffer (10 mM Tris-HCl (pH7.8), 150 mM NaCl, 0.5 mM EDTA, 0.5% Na.Deoxycholate, 0.5% NP-40, 1 mM PMSF, 1mM DTT, cOmplete^TM^, EDTA-free Protease Inhibitor Cocktail (Roche, Basil, Switzerland)) was added and the sample mixed by pipetting up and down. Samples were incubated on ice for 30 min with pipetting every 10 min, after which they were centrifuged at 18,000 *g* for 15 min at 4 °C. Supernatants were transferred to new tubes and used for immunoprecipitation with GFP-Trap^®^ Agarose beads (Chromotek, Munich, Germany) according to the manufacturer’s recommendations. 

Individual protein samples were processed by filter-aided sample preparation (FASP) method3 with some modifications as specified. Following IP washes, bead bound protein complexes were mixed with 2% SDS solution and heated to 50 °C for 25min after which the supernatant is reduced using DTT at 95 °C. After cooling to RT, samples are loaded onto the Microcon device with MWCO 30 kDa (Merck Millipore, Darmstadt, Germany) and centrifuged at 7000 × g for 30 min at 20 °C. The retained proteins were washed (all centrifugation steps after sample loading done at 14,000 × g) with 200 μL UA buffer. The final protein concentrates kept in the Microcon device were mixed with 100 μL of UA buffer containing 50 mM iodoacetamide and incubated in the dark for 20 min. After the next centrifugation step, the samples were washed three times with 100 μL UA buffer and three times with 100 μL of 50 mM NaHCO3. Trypsin (0.75 μg, sequencing grade, Promega) was added onto the filter and the mixture was incubated for 18 h at 37 °C. The tryptic peptides were finally eluted by centrifugation followed by two additional elutions with 50 μL of 50mM NaHCO_3_. Peptides were then cleaned by liquid-liquid extraction (3 iterations) using water saturated ethyl acetate2. Cleaned FASP eluate is evaporated completely in SpeedVac concentrator (Thermo Fisher Scientific, Waltham, MA, USA). Resulting peptides were extracted into LC-MS vials by 2.5% formic acid (FA) in 50% acetonitrile (ACN) and 100% ACN with addition of polyethylene glycol (20,000; final concentration 0.001%)1 and concentrated in a SpeedVac concentrator (Thermo Fisher Scientific, Waltham, MA, USA).

### 4.10. LC/MS Analysis of Peptides

LC-MS/MS analyses of all peptide mixtures were performed using RSLCnano system connected to Orbitrap Fusion Lumos mass spectrometer (Thermo Fisher Scientific, Waltham, MA, USA). Prior to LC separation, tryptic digests were online concentrated and desalted using trapping column (300 μm × 5 mm, μPrecolumn, 5μm particles, Acclaim PepMap100 C18, Thermo Fisher Scientific; temperature of 40 °C). After washing of trapping column with 0.1% FA, the peptides were eluted (flow rate −300 nL/min) from the trapping column onto an analytical column (Acclaim Pepmap100 C18, 3 µm particles, 75 μm × 500 mm; at temperature of 25 °C, (Thermo Fisher Scientific, Waltham, MA, USA) by 75 min linear gradient program (1–40% of mobile phase B; mobile phase A: 0.1% FA in water; mobile phase B: 0.1% FA in 80% ACN). Equilibration of the trapping column and the analytical column was done prior to sample injection to sample loop. The analytical column outlet was directly connected to the Digital PicoView 550 (New Objective) ion source with sheath gas option and SilicaTip emitter (New Objective; FS360–20–15-N-20-C12) utilization. ABIRD (Active Background Ion Reduction Device, ESI Source Solutions) was installed.

MS data were acquired in a data-dependent strategy with cycle time for 2 s and with survey scan (m/z 350–2000). The resolution of the survey scan was 60,000 (at m/z 200) with a target value of 4 × 10^5^ ions and maximum injection time of 50 ms. HCD MS/MS (30% relative fragmentation energy, normal mass range) spectra were acquired with a target value of 5.0 × 10^4^ and resolution of 30 000 (at m/z 200. The maximum injection time for MS/MS was 500 ms. Dynamic exclusion was enabled for 60 s after one MS/MS spectra acquisition. The isolation window for MS/MS fragmentation was set to 1.6 m/z.

The analysis of the mass spectrometric RAW data files was carried out using the MaxQuant software (version 1.6.10.43) using default settings unless otherwise noted. MS/MS ion searches were done against modified cRAP database (based on http://www.thegpm.org/crap) containing protein contaminants like keratin, trypsin, etc., and UniProtKB protein database for Arabidopsis thaliana (ftp://ftp.uniprot.org/pub/databases/uniprot/current_release/knowledgebase/reference_proteomes/Eukaryota/UP000006548_3702.fasta.gz; downloaded 05.2020, version 2020/05, number of protein sequences: 27,463). Oxidation of methionine and proline, deamidation (N, Q) and acetylation (protein N-terminus) as optional modification, and carbamidomethylation (C) as fixed modification. Trypsin/P enzyme with 2 allowed missed cleavages were set. Peptides and proteins with FDR threshold < 0.01 and proteins having at least one unique or razor peptide were considered only. Match between runs was set among all analyzed samples. Protein abundance was assessed using protein intensities calculated by MaxQuant.

Protein intensities reported in proteinGroups.txt file (output of MaxQuant) were further processed using the software container environment (https://github.com/OmicsWorkflows), version 3.7.2a. Processing workflow is available upon request. Briefly, it covered: a) removal of decoy hits and contaminant protein groups, b) protein group intensities log2 transformation, c) LoessF normalization, and d) differential expression using LIMMA statistical test.

### 4.11. Yeast Two-Hybrid Assays

The yeast strain MaV203 (MATα, leu2-3,112, trp1-901, his3∆200, ade2-101, gal4∆, gal80∆, SPAL10_UASGAL1_::URA3, GAL1::lacZ, HIS3_UASGAL1_::HIS3@LYS2, can1^R^, cyh2^R^) (Vidal, 1997) was transformed as described [13]. First, negative auto-activation of the full length 14–3–3 ε bait protein was confirmed before testing in combination with respective preys. For Yeast two-hybrid assays, MaV203 was co-transformed with respective bait and prey plasmids and grown on synthetic complete media lacking leucine and tryptophan (SC-L/-T) selection plates. Interaction was screened on synthetic complete media lacking leucine, tryptophan, histidine, and added 10 mM 3-AT (3-Amino-1,2,4-triazole) (SC-L/−T/−H/+10mM 3AT). Briefly, colonies growing on SC-L/-T plates were resuspended in water to an OD_600_ of 0.5 and serially diluted (5 × dilution factor). Five µl of each dilution was spotted on SC-L/−T/−H/+10mM 3AT and incubated at 28 °C for 5 days. Transformation and interaction tests were repeated four times. 

### 4.12. RNA-Seq Library Preparation and Sequencing

Total RNA was isolated from 1-week-old Col-0 wt and *bzip* mutant seedlings (up to 100 mg) using the RNeasy Plant Mini kit (Qiagen, Hilden, Germany). Isolated RNA was treated with DNase (Thermo Fischer, Ambion, Austin, TX, USA) and RNA integrity (RIN) analyzed using a Bioanalyzer (Agilent, Santa Clara, CA, USA). RNA (2 μg) with a RIN >7 was subsequently used for strand specific cDNA library preparation by Eurofins (Eurofins Genomics GmbH, Ebersberg, Germany). Samples were sequenced on NovaSeq 6000 S2 PE150 XP Illumina sequencing machine (Illumina, San Diego, CA, USA) by Eurofins.

The analysis resulted in approximately 36 million of 151 bp long paired-end reads per sample. Raw reads were subjected to quality control (phred score > 20) and adapter trimming using FastQC (0.11.8), Cutadapt (v. 1.9.1), and Trim Galore! (v. 0.6.5) [54,55,56]. Resulting reads were mapped to the TAIR 10 genome with STAR aligner, version 2.7.5a [57] using default parameters for paired-end data. The featureCounts program from the Subread package, version 1.6.2 [58] was used to generate the count matrix. Differentially expressed genes were selected using DeSeq2 packages in R [59,60]. After independent filtering of the results, transcripts with adjusted *p*-value < 0.05 and log2 fold change >1 (upregulated) or < −1 (downregulated) were considered to be significantly differentially expressed. Gene ontology analysis (statistical overrepresentation test) was done using Panther Classification System [61]. Gene ontology analysis was performed using the ShinyGO v0.61 (http://bioinformatics.sdstate.edu/go, visited 26 October 2020). The data were deposited into BioProject ID PRJNA681197 (https://www.ncbi.nlm.nih.gov/bioproject/589533).

### 4.13. Chromatin Immunoprecipitation Sequencing

Chromatin Immunoprecipitation (ChIP) was performed according to [62] with minor modifications. Briefly, 1.5 g of three-week-old seedlings expressing bZIP-GFP fusion proteins (pGWB5) were cut in smaller pieces and crosslinked with 1% formaldehyde on ice for 10 min under a vacuum. The formaldehyde was quenched with 0.125 M glycine for 10 min. Samples were frozen in liquid nitrogen and crushed to a powder. Nuclei were extracted with Honda buffer (2.5 % *w*/*v* Ficoll 400, 5% *w*/*v* dextran T40, 0.4 M sucrose, 25 mM Tris-HCl-pH 7.4, 10 mM MgCl2, 10 mM β-mercaptoethanol, 0.5% Triton X-100 and 1 × Protease Inhibitor-P9599, Sigma-Aldrich) and filtered first through Miracloth and then through 50 μm filter. Clean nuclei were lysed in lysis buffer (50 mM Tris-HCl-pH 8.0, 10 mM EDTA, 1% SDS, 1 × Protease Inhibitor-P9599, Sigma-Aldrich). Chromatin was diluted 10× (1.1% Triton X-100, 1.2 mM EDTA, 16.7 mM Tris-HCl-pH 8.0, 167 mM NaCl, 1 × Protease Inhibitors-P9599, Sigma-Aldrich) and fragmented with Sonopuls HD 2070 uL-trasonic homogenizer (Bandelin, Berlin, Germany) with the following settings: 360 s, 60% duty cycle, and 50% power. For the immunoprecipitation, 30 µL of GFP-Trap beads (Chromotek, Munich, Germany) were used with overnight incubation. Only the Low Salt Wash buffer (150 mM NaCl, 0.1% SDS, 1% Triton X-100, 2 mM EDTA and 20 mM Tris-HCl-pH 8.0) was used to wash the beads. Decrosslinking and elution were done with the IPure Kit following manufacturer’s instructions (Cat. No. C03010015, Diagenode, Liège, Belgium). The isolated DNA was used for library preparation using NEBNext^®^ Ultra™ II DNA Library Prep Kit for Illumina^®^ (Cat No. E7645) according to the manufacturer’s protocol. The library was then used for paired-end sequencing on Illumina NovaSeq platform by Novogene (Beijing, China). The data were deposited into BioProject ID PRJNA681356 (https://www.ncbi.nlm.nih.gov/bioproject/589533).

The quality control of the data was performed by FastQC (v0.72). Reads were trimmed using Skewer (Parameters: 0.1.126) and low quality reads were discarded (i.e., reads with proportion of low quality bases larger than 50%, N ratio larger than 15%, reads with adaptor at the 5′-end, reads without adaptor and inserted fragment at the 3′-end, and reads shorter than 18nt after trimming). The reads were mapped to the TAIR10 reference genome of Arabidopsis using BWA [63]. MACS2 (v 2.1.1) was used to identify enriched regions using default parameters and effective genome size of 1.19e+08. The three replicates were used together and the samples from negative control (samples from the free GFP expressing plants) were used as control file [64]. DiffBind (v 2.10.0) was used to calculate differences between samples with FDR treshhold 0.05 [65]. ChIPseeker (v1.18.0) was used to annotate the identified peaks [66] with Araport11 annotation set and BioVenn to compare the sets of annotated genes [67]. Hypergeometric distribution function was used to estimate significance of overlaps between gene sets. Only nuclear genome was used for further analyses. The coverage profiles were visualized by deepTools (v3.3.2), with the .bw files scaled to number of reads per bin with scaling factor for 1x average coverage for the samples and 0.5 × for the negative control (to achieve same background baseline when the coverage is plotted for all genes at TSS). The motif analyses was done with MEME-ChIP (v5.2.0) on 500 bp regions centered on summits outputted from MACS2 [68]. Gene ontology analysis was performed using the ShinyGO (v0.61) [69]. Some of the analyses were done using the Galaxy platform at galaxy.metacentrum.cz [70].

## 5. Conclusions

In conclusion, we have shown that a portion of bZIP transcription factors, bZIP18 and bZIP52, are sequestered to the cytoplasm through binding to 14–3–3 proteins. Following heat stress, bZIP proteins become dephosphorylated, allowing them to dissociate from 14–3–3 proteins and translocate to nuclei. There, they jointly regulate the expression of numerous genes. In seedlings, both bZIP18 and bZIP52 seem to act predominantly as repressors, since their mutation caused the upregulation of almost twice as many differentially expressed genes than their downregulation. Interestingly, the set of downregulated genes comprised a more organized set of genes, predominantly involved in energy metabolism, stress response, and translation. On top of direct transcriptional regulation, these bZIP TFs are likely to regulate gene expression after stress treatment also via modulation of lncRNA expression. The metabolic pathways affected and the precise regulation mechanism in relation to heat stress treatment need to be explored in more detail, and are subject to further investigation.

## Data Availability

The data presented in this study are openly available in BioProject, with ID PRJNA681356 for ChIP-seq data and ID PRJNA681197 for RNA-seq data at https://www.ncbi.nlm.nih.gov/bioproject/589533.

## Supplementary Materials

Supplementary materials can be found at https://www.mdpi.com/1422-0067/22/2/530/s1.

## Abbreviations

HS	Heat stress
TF	Transcription factor
HSR	Sheat stress response
HSF	heat shock transcription factors
bZIP	basic leucine zipper
ER	Endoplasmic reticulum
UPR	Unfolded protein response
IRE1	INOSITOL REQUIRING ENZYME 1
ABA	Abscisic acid
LEA	Late embryogenesis abundant
VIP1	VirE2-interacting protein 1
GFP	Green Fluorescent Protein
DAPI	4′,6-diamidino-2-phenylindole dihydrochloride
OA	Okadaic Acid
PP2A	Protein Phosphatase 2A
LC-MS/MS	Liquid Chromatography tandem Mass Spectrometry
Y2H	Yeast 2 Hybrid
BiFC	Bimolecular Fluorescence Complementation
BD	Binding Domain
AD	Activation Domain
3AT	3-Amino-1,2,4-triazole
YFP	Yellow Fluorescent Protein
WT	Wild Type
PCA	Principal Component Analysis
DEGs	Differentially Expressed Genes
GO	Gene Ontology
lncRNA	long non-coding RNA
OX	Over-expression
TSS	Transcription start site
MS	Murashige and Skoog
