# Supplementary material for: Arabidopsis bZIP18 and bZIP52 Accumulate in Nuclei Following Heat Stress where They Regulate the Expression of a Similar Set of Genes
Source: Int J Mol Sci. 2021 Jan 7;22(2):530. doi: 10.3390/ijms22020530 (PMC7830406; doi:10.3390/ijms22020530)
Supplement: Supplementary file 1 [file ijms-22-00530-s001.zip › ijms-1040585-proofback-suppl/ijms-1040585-proofback-Supplementary Tables/Supplementary Tables/Supplementary Table Legends.docx]

Supplementary Table Legends:

**Supplementary Table S1. Putative 14-3-3 interacting partners identified through pull-down assays in *A. thaliana* seedlings.** Proteins identified as potential interacting partners are shown in table format when bZIP18-GFP and bZIP52-GFP were used as baits. Only those proteins with a minimum of two significant peptides are listed, one of which was unique. Total number of replicates was two.

**Supplementary Table S2.** **Quality of RNA and total number of reads per biological replicate.**

**Supplementary Table S3. Normalized expression values.**

**Supplementary Table S4. Table of all significantly differentially expressed genes in each comparison*.*** The table consists of 8 columns: Gene ID, base mean, fold change in log_2_ scale, p-value, adjusted p-value, symbol of gene, length of gene and brief description obtained from Araport database. Genes are sorted according to their log_2_Fold Change.

**Supplementary Table S5. Matrix of Deseq2 size factor normalized read counts of all RNAseq samples.**

**Supplementary Table S6. Summary of raw data quality control, mapping and strand cross correlation.**

**Supplementary Table S7. List of genes that were identified as enriched either for bZIP18, bZIP52 or bZIP18xbZIP52.**

**Supplementary Table S8. 30 most significant Gene Ontology (GO) terms that were found among genes nriched either for bZIP18, bZIP52 or bZIP18x52.**

**Supplementary Table S9. List of primers used in this study**.
