# Supplementary material for: Arabidopsis bZIP18 and bZIP52 Accumulate in Nuclei Following Heat Stress where They Regulate the Expression of a Similar Set of Genes
Source: Int J Mol Sci. 2021 Jan 7;22(2):530. doi: 10.3390/ijms22020530 (PMC7830406; doi:10.3390/ijms22020530)
Supplement: Supplementary file 1 [file ijms-22-00530-s001.zip › ijms-1040585-proofback-suppl/ijms-1040585-suppl.docx]

**Supplementary Table S1.** Putative 14–3–3 interacting partners identified through pull-down assays in *A. thaliana* seedlings. Proteins identified as potential interacting partners are shown in table format when bZIP18-GFP and bZIP52-GFP were used as baits. Only those proteins with a minimum of two significant peptides are listed, one of which was unique. Total number of replicates was two. **Supplementary Table S2.** Quality of RNA and total number of reads per biological replicate. **Supplementary Table S3.** Matrix of Deseq2 size factor normalized read counts of all RNAseq samples. **Supplementary Table S4.** Table of all significantly differentially expressed genes in each comparison*.* The table consists of 8 columns: Gene ID, base mean, fold change in log_2_ scale, *p*-value, adjusted *p*-value, symbol of gene, length of gene, and brief description obtained from Araport database. Genes are sorted according to their log_2_Fold Change. **Supplementary Table S5.** Summary of raw data quality control, mapping, and strand cross correlation for ChIP-seq samples. **Supplementary Table S6.** List of genes that were identified as enriched either for bZIP18, bZIP52, or bZIP18xbZIP52. **Supplementary Table S7.** Thirty most significant Gene Ontology (GO) terms that were found among genes enriched for bZIP18, bZIP52, and bZIP18xbZIP52. **Supplementary Table S8.** List of primers used in this study.

**Supplementary Figure Legends:**

**Supplementary Figure S1.** bZIP18 and bZIP52 do not possess transmembrane domains. Amino acid sequences of bZIP18, bZIP52, and bZIP60 were analyzed using the TMHMM-2.0 software (http://www.cbs.dtu.dk/services/TMHMM/) [1]. Red lines indicate transmembrane regions of a protein, a feature present in bZIP60, but absent in bZIP18 and bZIP52. **Supplementary Figure S2.** Localization of free eGFP. As a negative control for localization studies in *N. benthamiana,* free GFP was co-expressed in the same system and localization of free GFP investigated alongside HDEL-mCherry (ER marker). **Supplementary Figure S3.** Interaction analyses of bZIP18 and bZIP52 with 14–3–3 ε (Figure 5B), all channels. BiFC analysis of bZIP variants against 14–3–3 ε. nYFP-fused 14–3–3 ε was co-expressed with either cYFP-fused bZIP18 or bZIP52 in *N. benthamiana* epidermal cells, and YFP signals detected using confocal microscopy. YFP reconstitution (positive interaction) was observed for both single mutant variants of each protein. Only when both residues were mutated (bZIP18-S39A/S120A and bZIP52-S40A/S117A), no YFP signal was detected. **Supplementary Figure S4.** bZIP18 and bZIP52 form heterodimers. (A) Protein-protein interactions evaluated by BiFC assays. cYFP-fused bZIP18 was co-expressed with either nYFP-fused bZIP52 or ATA20 (negative control) in *N. benthamiana* epidermal cells, and YFP signals detected using confocal microscopy. Reconstitution of the YFP fluorophore indicates a positive interaction, while RFP serves as a transformation control. YFP reconstitution was observed when bZIP18 and bZIP52 were. (B) Protein-protein interactions evaluated by yeast two hybrid. Baits were expressed as GAL4 DNA-binding domain (BD) fusions and prey as GAL4 activation domain (AD) fusions. Bait and prey constructs were co-transformed into MAV203, and colonies spotted onto control (−L/−T) and phenotyping (−L/−T/−H/+10 mM 3AT) plates. Plates were incubated at 28 °C for 2 days prior to taking photos. When bZIP18 and bZIP52 were co-expressed, yeast growth was observed on media lacking histidine, indicating positive interactions. **Supplementary Figure S5.** Pearson correlation matrix showing similarity between individual ChIP-seq samples. The scores were calculated based on mapped read coverage data. Each sample type has three biological replicates; the replicates are denoted by the number after underscore. **Supplementary Figure S6.** Coverage of ChIP-seq reads at enriched regions. The regions enriched either for bZIP18, bZIP52, or bZIP18x52 are in rows. The samples mapped to these regions are in columns. Each sample consists of three biological replicates. **Supplementary Figure S7.** DiffBind MA plots showing the differences between samples. Regions marked with magenta significantly differ between samples. **Supplementary Figure S8.** Coverage of ChIP-seq reads at transcription start sites (TSS) and adjacent regions over all genes in *A. thaliana* (Araport11 annotation) showed as average profile and heatmap. Each sample consists of three biological replicates. **Supplementary Figure S9.** Coverage of ChIP-seq reads at transcription start sites (TSS) and adjacent regions of enriched genes. The genes associated with regions enriched either for bZIP18, bZIP52, or bZIP18x52 are in rows. The samples mapped to these genes are in columns. Each sample consists of three biological replicates. **Supplementary Figure S10.** Association of enriched regions with genomic elements. The graphs show percentage of regions enriched either for bZIP18, bZIP52, or bZIP18x52 that overlap given genomic element. Each sample consists of three biological replicates. **Supplementary Figure S11.** Three most abundant motifs discovered at regions enriched either for bZIP18, bZIP52, or bZIP18x52. The last column shows top five matches in the ArabidopsisDAPv1 database (O’Malley et al. 2016).

**Other supplementary materials:**

**Supplementary_File_S1.tar.gz.** Output of the MEME-ChIP tool for common regions enriched for bZIP18, bZIP52, and bZIP18x52. The output contains results of de novo motif discovery, search for known motifs, identification of secondary motifs, etc. For more details see: http://meme-suite.org/

Reference

1 Krogh, A.; Larsson, B.; Heijne, G.v.; Sonnhammer, E.L. Predicting transmembrane protein topology with a hidden Markov model: Application to complete genomes. *J. Mol. Biol.* **2001**, *305*, 567–580, doi:10.1006/jmbi.2000.4315.
