# Supplementary figures and images for: Arabidopsis bZIP18 and bZIP52 Accumulate in Nuclei Following Heat Stress where They Regulate the Expression of a Similar Set of Genes
Source: Int J Mol Sci. 2021 Jan 7;22(2):530. doi: 10.3390/ijms22020530 (PMC7830406; doi:10.3390/ijms22020530)

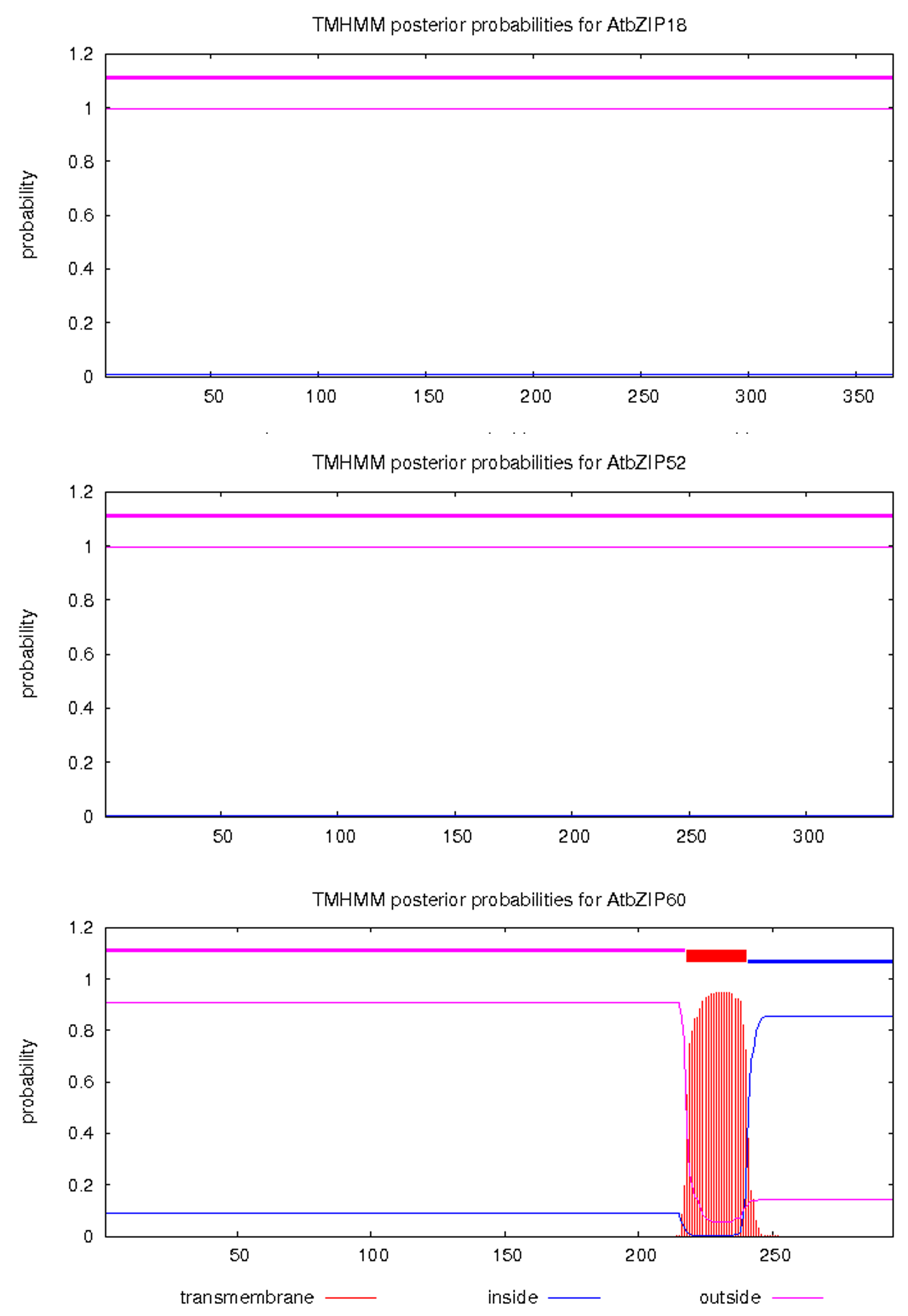

Supplement: Supplementary file 1 [file ijms-22-00530-s001.zip › ijms-1040585-proofback-suppl/ijms-1040585-proofbackSupplementary Figures/Supplementary Figures/Supplementary_Figure_S1.tif]

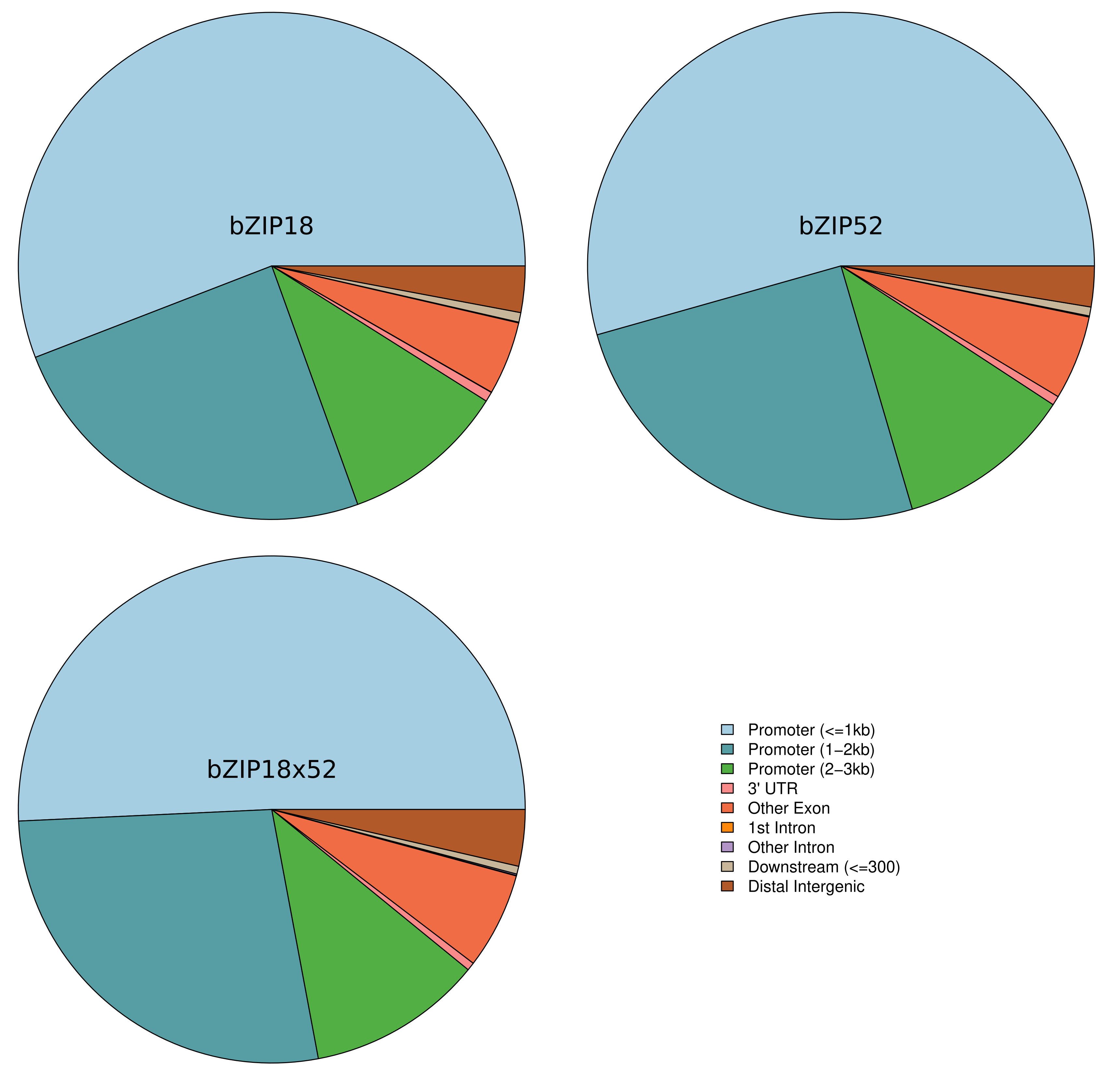

Supplement: Supplementary file 1 [file ijms-22-00530-s001.zip › ijms-1040585-proofback-suppl/ijms-1040585-proofbackSupplementary Figures/Supplementary Figures/Supplementary_Figure_S10.png]

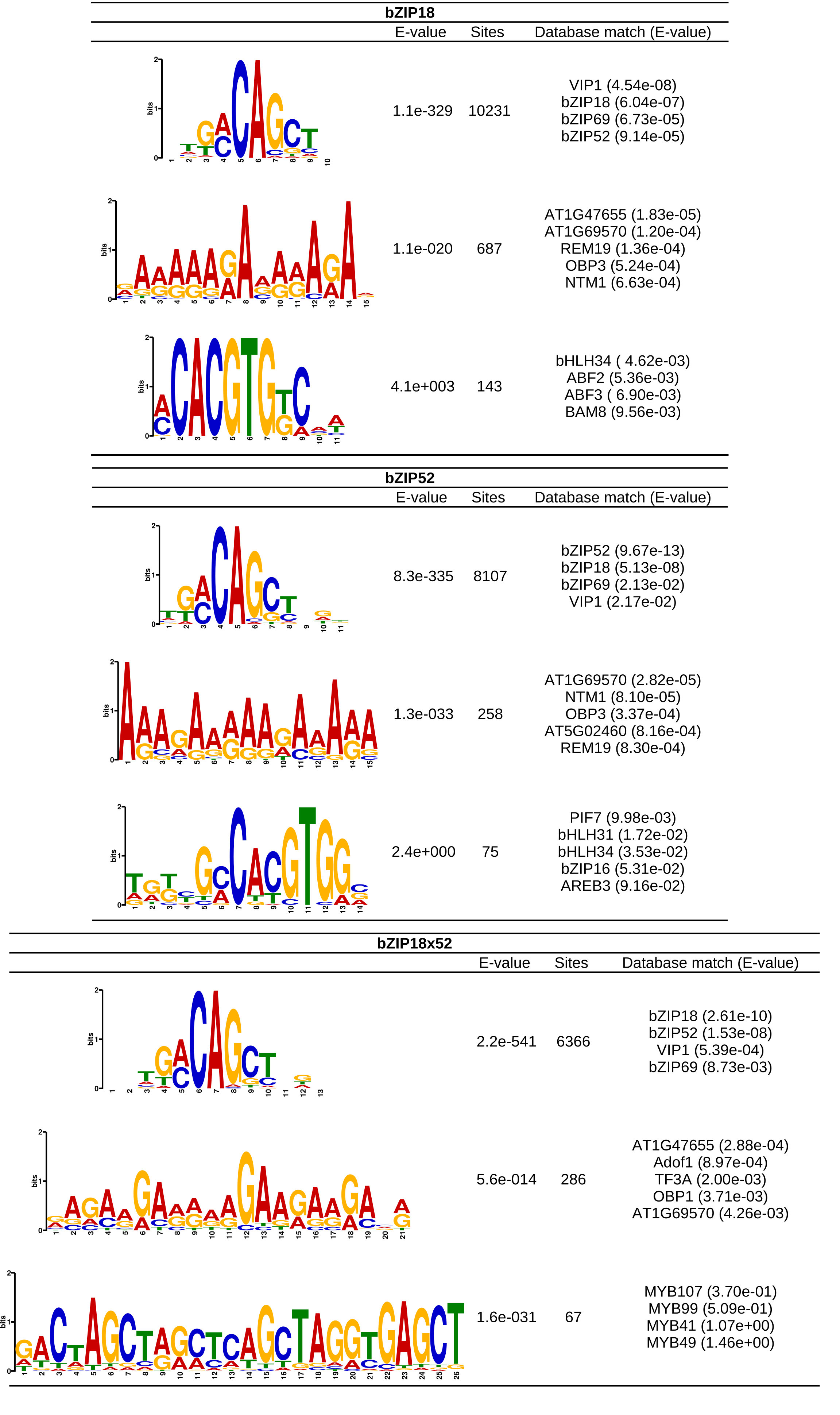

Supplement: Supplementary file 1 [file ijms-22-00530-s001.zip › ijms-1040585-proofback-suppl/ijms-1040585-proofbackSupplementary Figures/Supplementary Figures/Supplementary_Figure_S11.png]

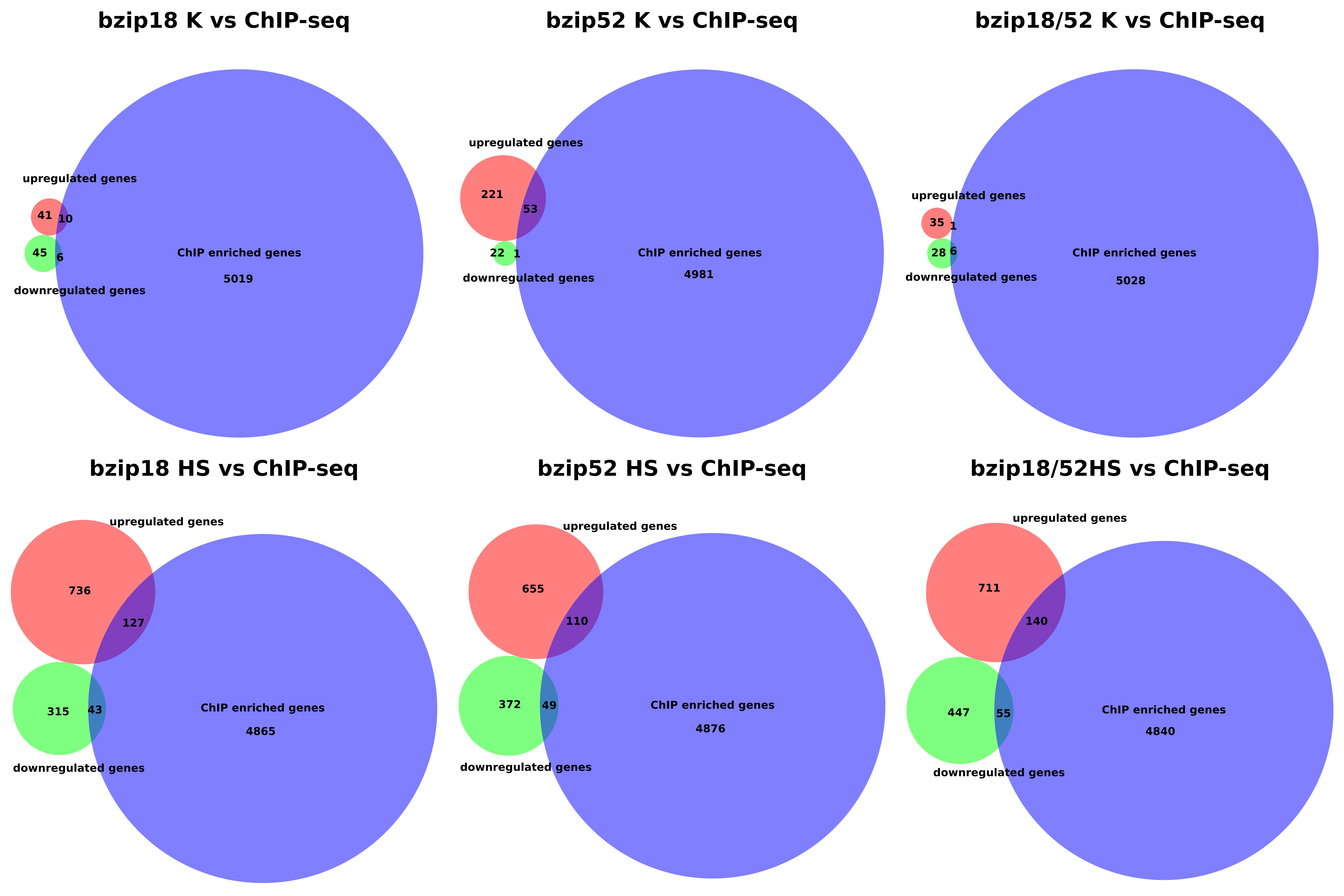

Supplement: Supplementary file 1 [file ijms-22-00530-s001.zip › ijms-1040585-proofback-suppl/ijms-1040585-proofbackSupplementary Figures/Supplementary Figures/Supplementary_Figure_S12.png]

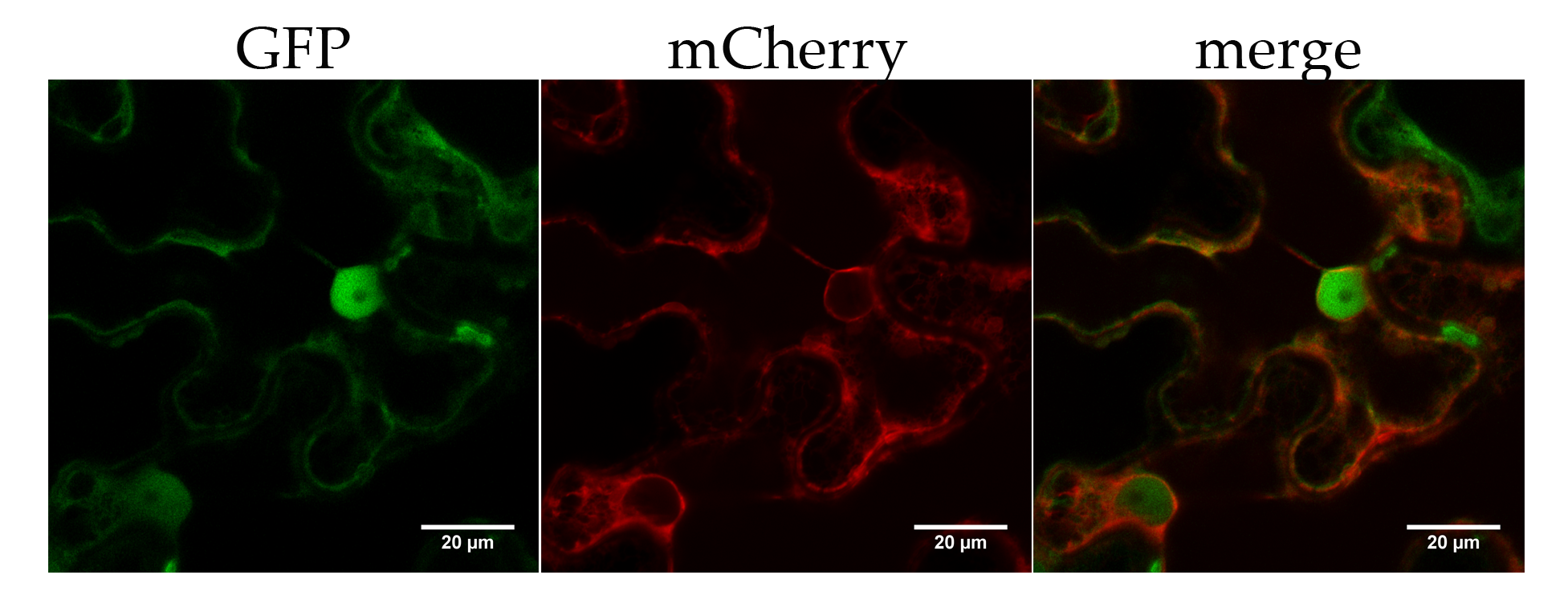

Supplement: Supplementary file 1 [file ijms-22-00530-s001.zip › ijms-1040585-proofback-suppl/ijms-1040585-proofbackSupplementary Figures/Supplementary Figures/Supplementary_Figure_S2.tif]

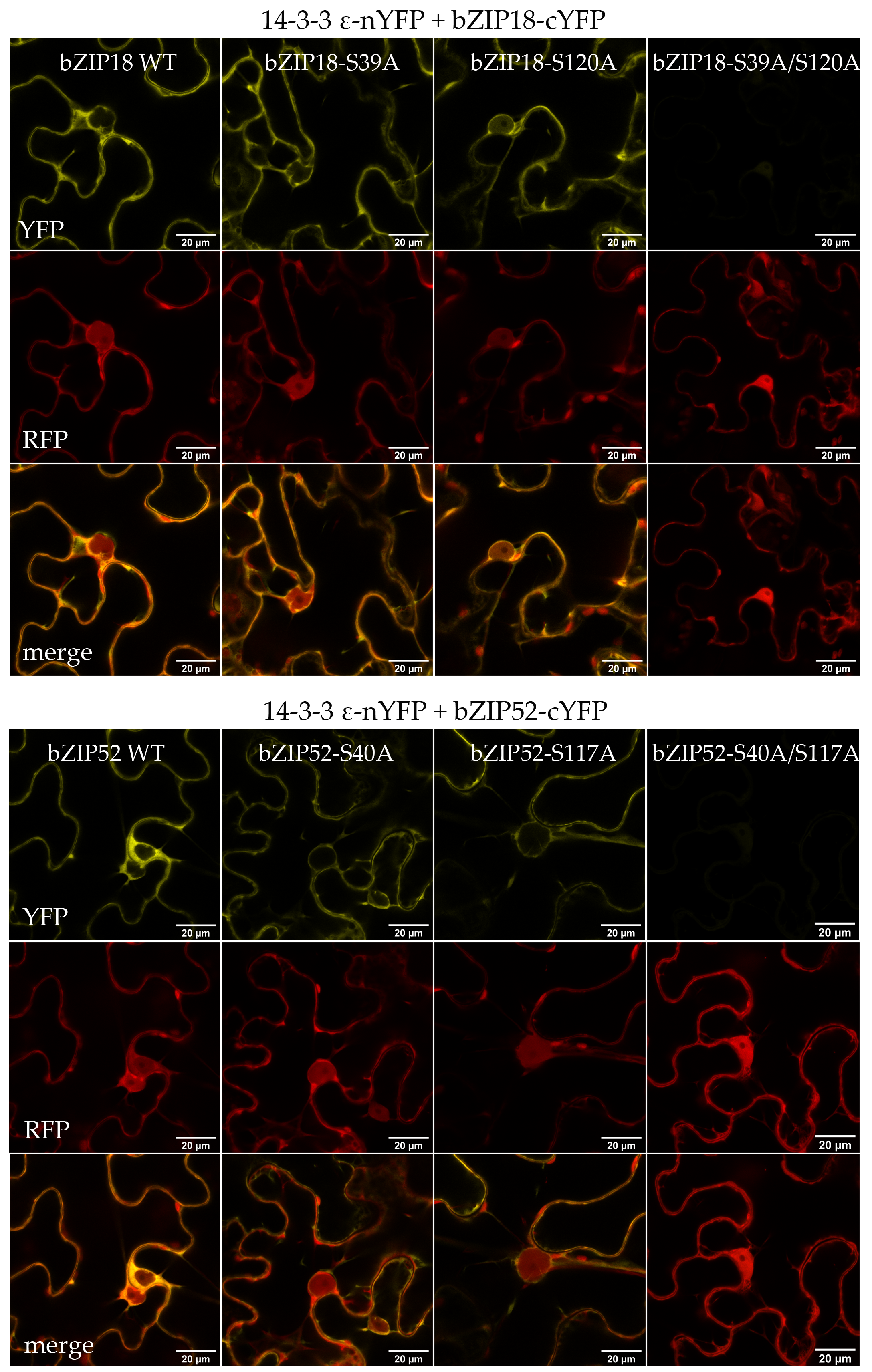

Supplement: Supplementary file 1 [file ijms-22-00530-s001.zip › ijms-1040585-proofback-suppl/ijms-1040585-proofbackSupplementary Figures/Supplementary Figures/Supplementary_Figure_S3.tif]

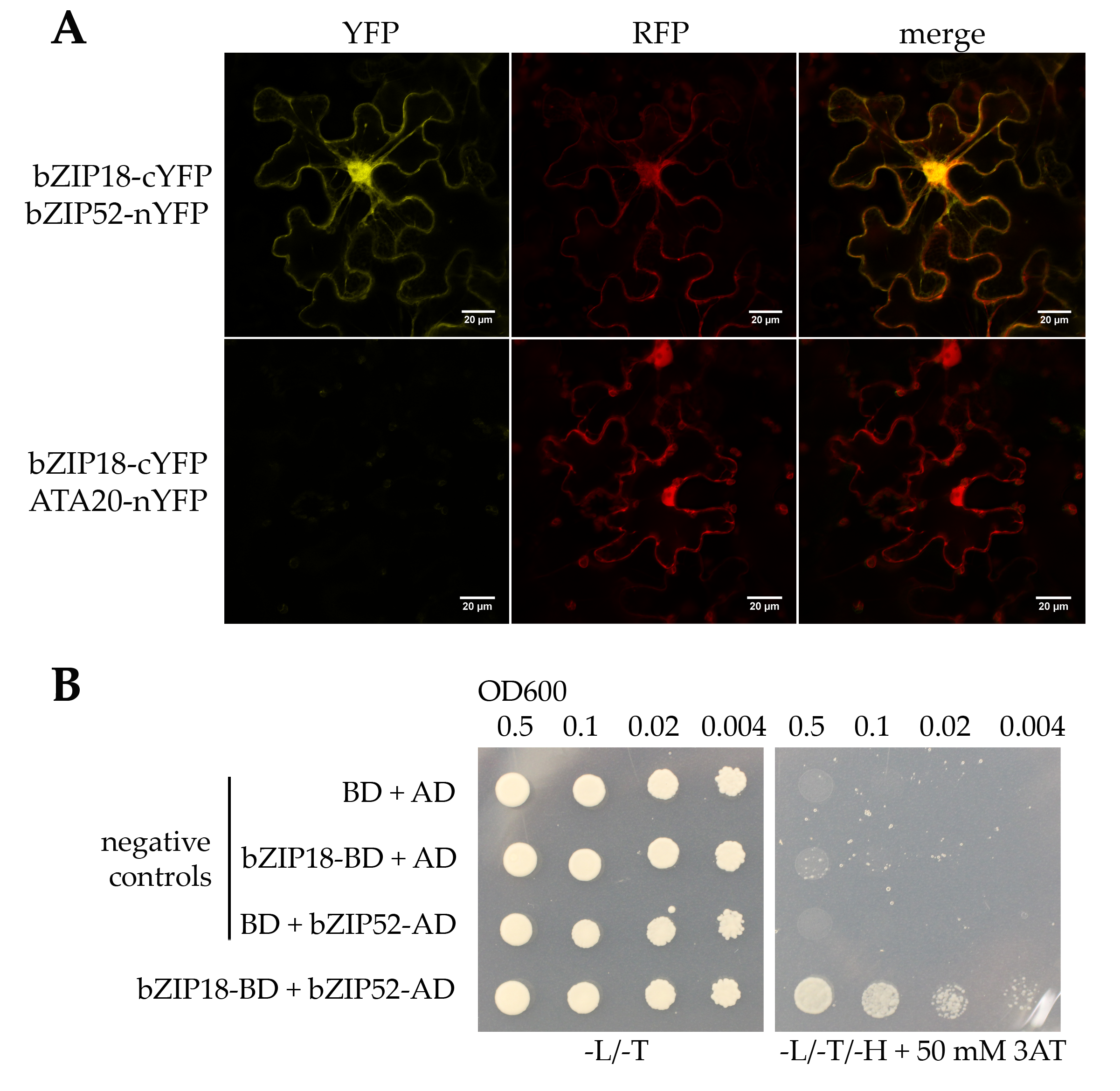

Supplement: Supplementary file 1 [file ijms-22-00530-s001.zip › ijms-1040585-proofback-suppl/ijms-1040585-proofbackSupplementary Figures/Supplementary Figures/Supplementary_Figure_S4.tif]

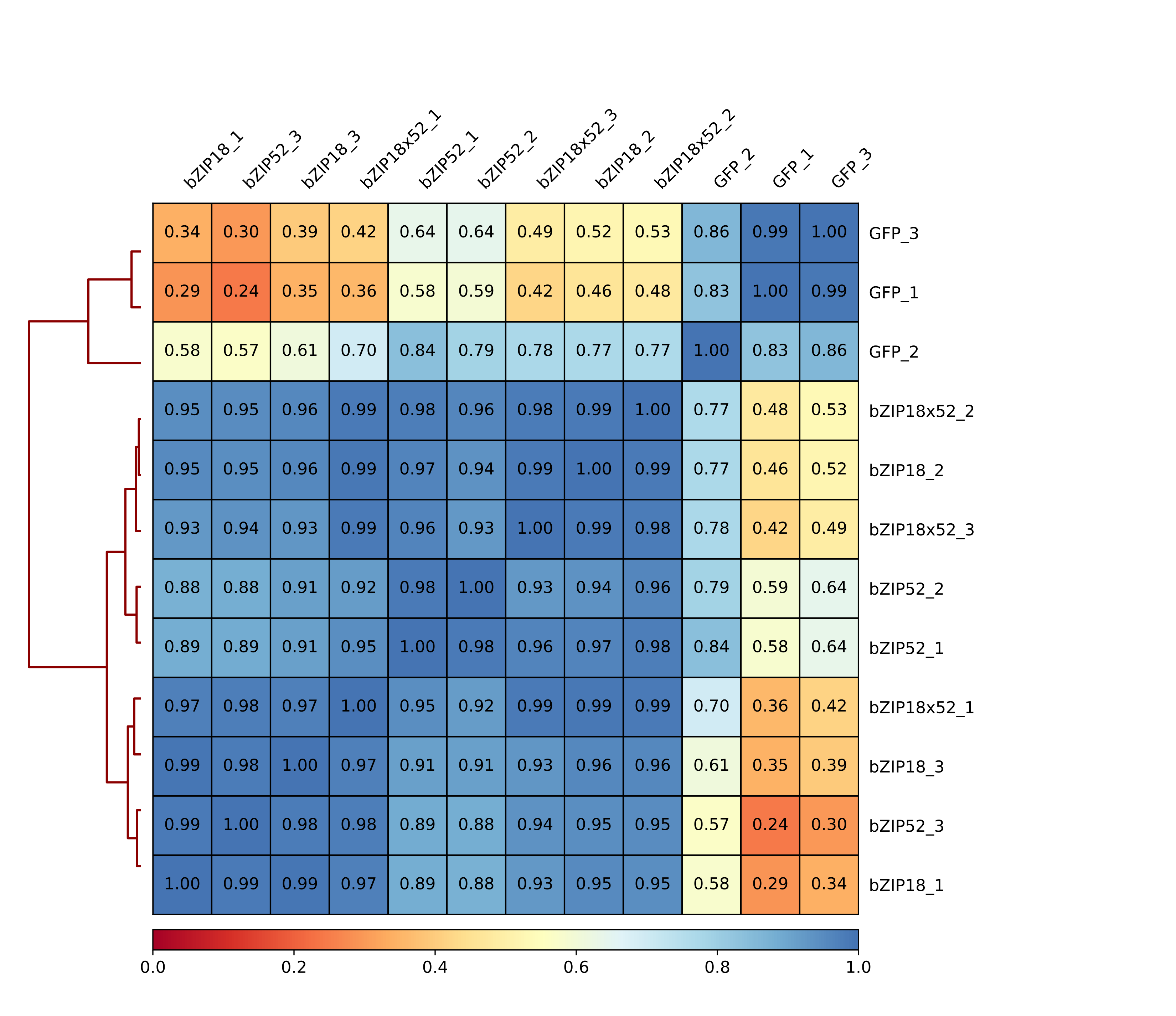

Supplement: Supplementary file 1 [file ijms-22-00530-s001.zip › ijms-1040585-proofback-suppl/ijms-1040585-proofbackSupplementary Figures/Supplementary Figures/Supplementary_Figure_S5.png]

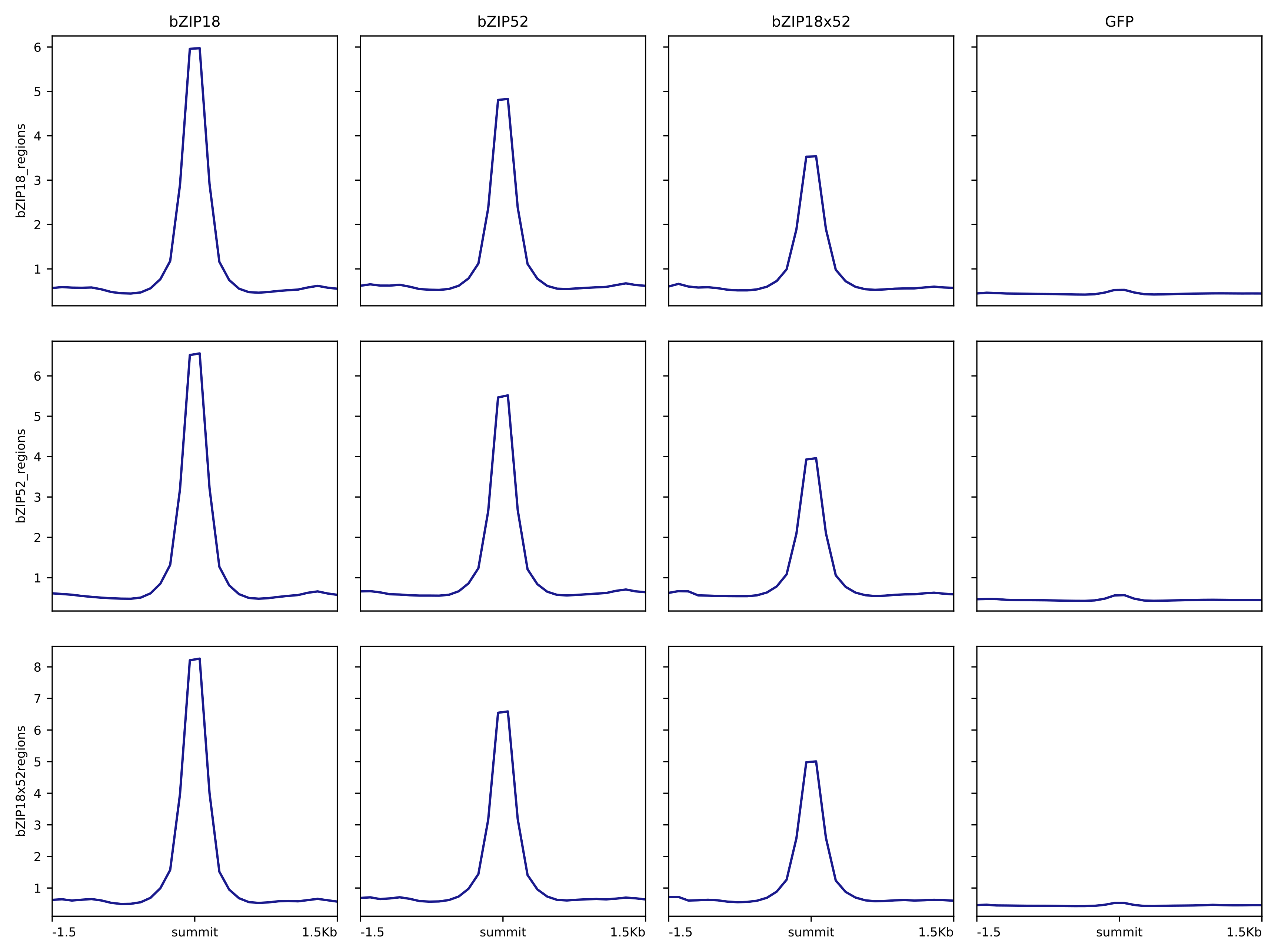

Supplement: Supplementary file 1 [file ijms-22-00530-s001.zip › ijms-1040585-proofback-suppl/ijms-1040585-proofbackSupplementary Figures/Supplementary Figures/Supplementary_Figure_S6.png]

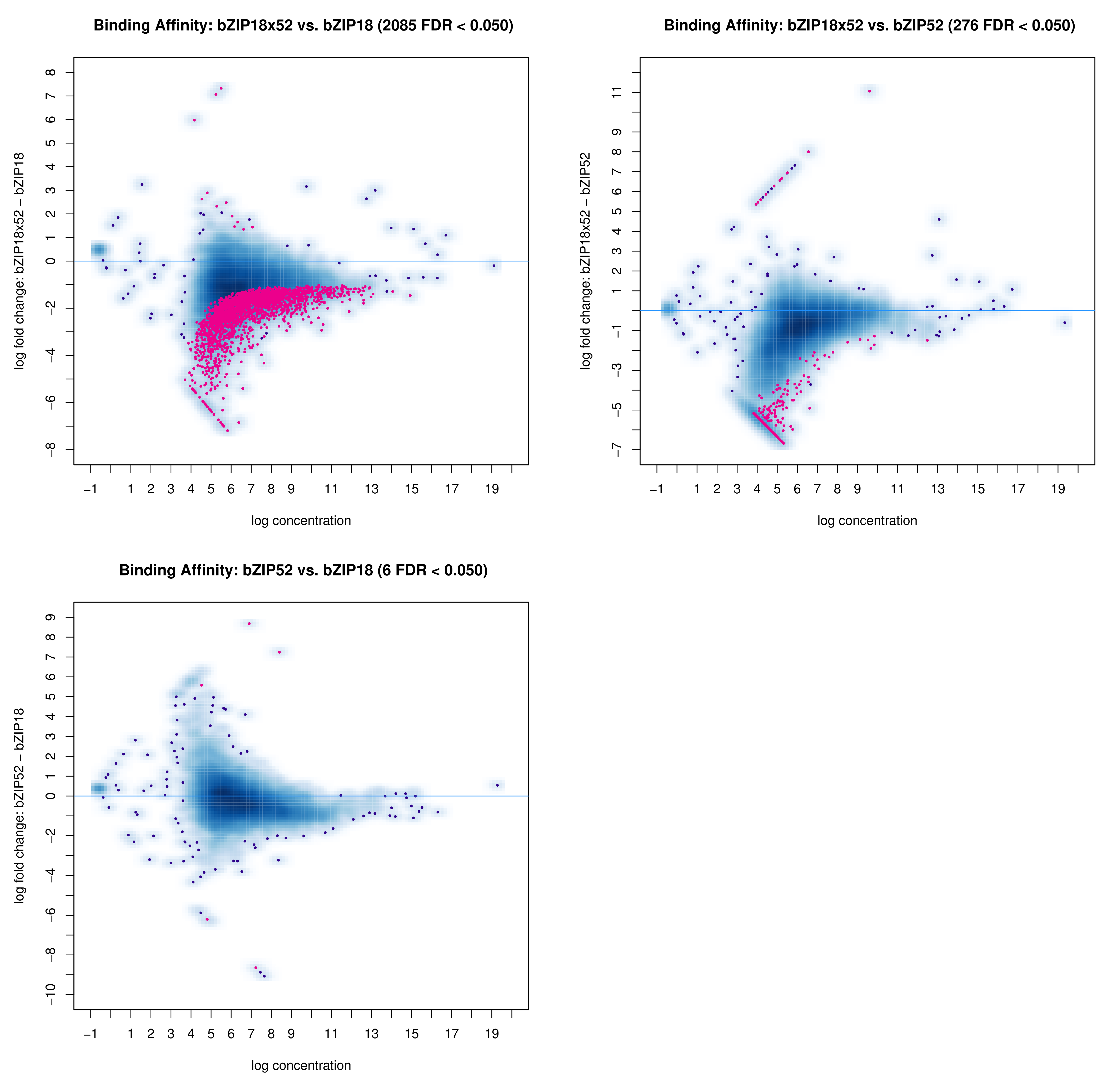

Supplement: Supplementary file 1 [file ijms-22-00530-s001.zip › ijms-1040585-proofback-suppl/ijms-1040585-proofbackSupplementary Figures/Supplementary Figures/Supplementary_Figure_S7.png]

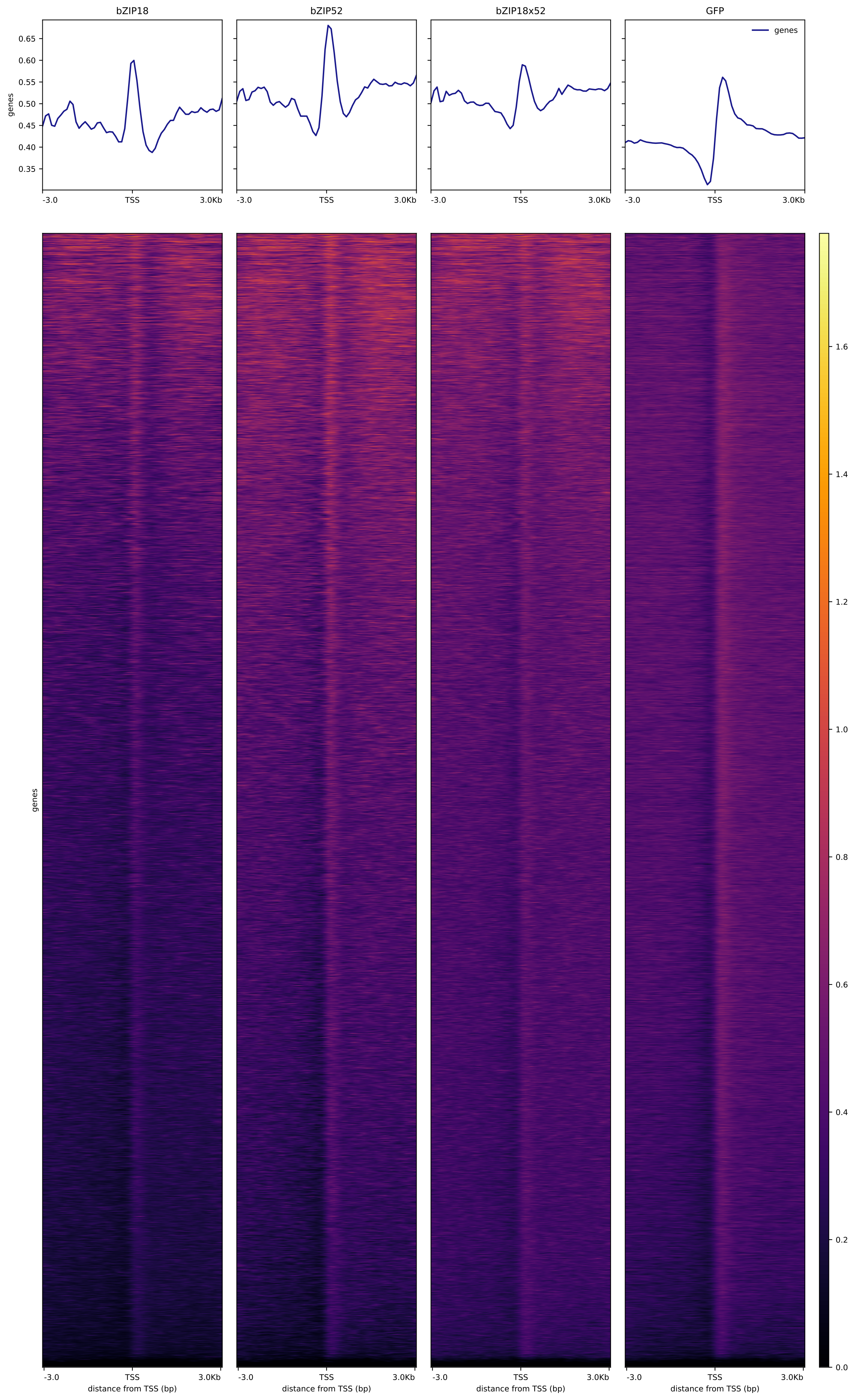

Supplement: Supplementary file 1 [file ijms-22-00530-s001.zip › ijms-1040585-proofback-suppl/ijms-1040585-proofbackSupplementary Figures/Supplementary Figures/Supplementary_Figure_S8.png]

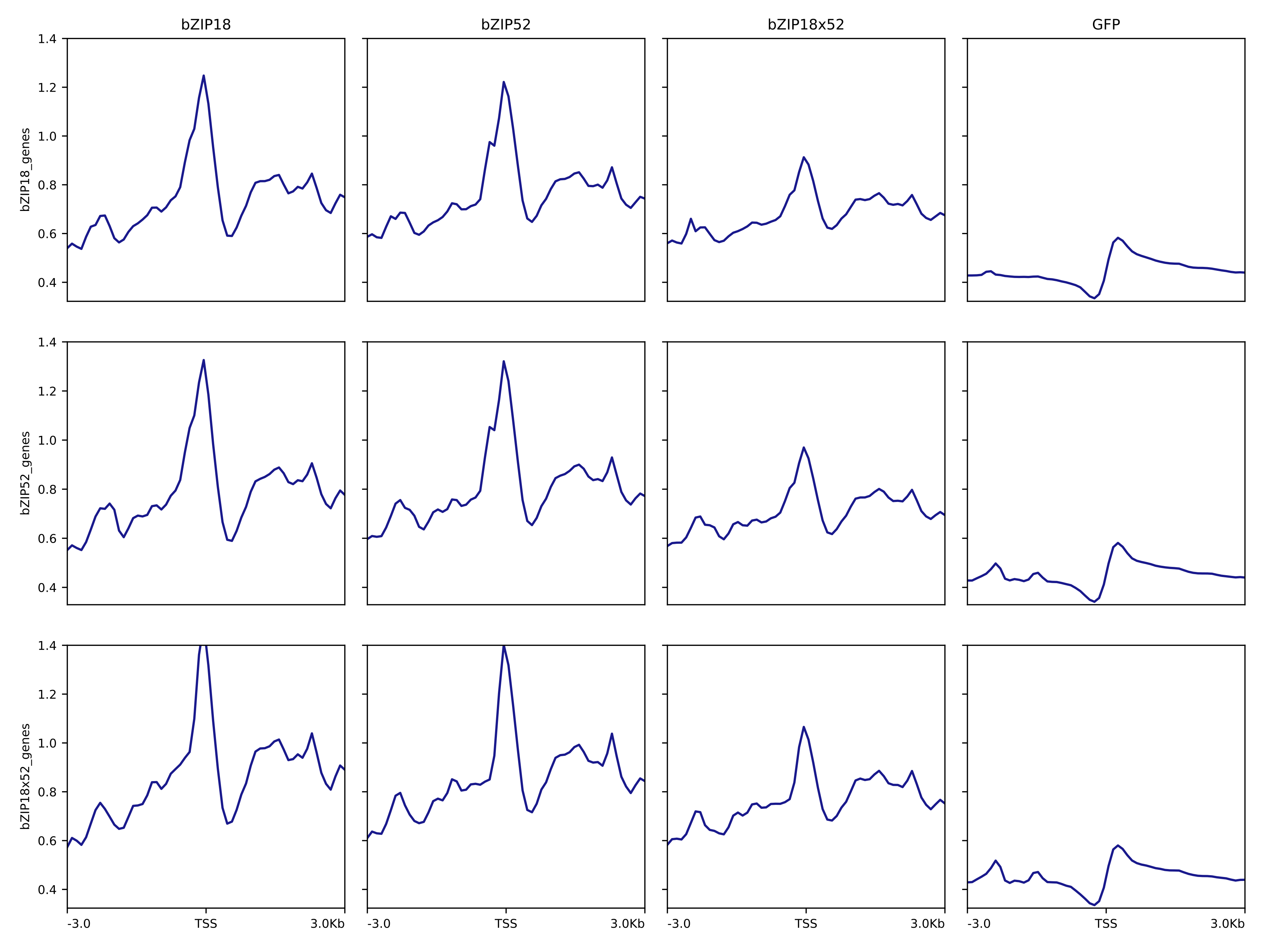

Supplement: Supplementary file 1 [file ijms-22-00530-s001.zip › ijms-1040585-proofback-suppl/ijms-1040585-proofbackSupplementary Figures/Supplementary Figures/Supplementary_Figure_S9.png]
