# Supplementary material for: Arabidopsis bZIP18 and bZIP52 Accumulate in Nuclei Following Heat Stress where They Regulate the Expression of a Similar Set of Genes
Source: Int J Mol Sci. 2021 Jan 7;22(2):530. doi: 10.3390/ijms22020530 (PMC7830406; doi:10.3390/ijms22020530)
Supplement: Supplementary file 1 [file ijms-22-00530-s001.zip › ijms-1040585-proofback-suppl/ijms-1040585-proofback-Supplementary Tables/Supplementary Tables/Supplementary_File_S1.tar.gz!/appMEMECHIP_5.2.016052793814261206909122/fimo_out_27/fimo.html]

FIMO Results


---

|  |  |  |  |  |
| --- | --- | --- | --- | --- |
| **Database and Motifs** | **High-scoring Motif Occurences** | **Debugging Information** | **Results in TSV Format** | **Results in GFF3 Format** |

  
  


---
